# Maternal fucosyltransferase 2 status affects the gut bifidobacterial communities of breastfed infants

**DOI:** 10.1186/s40168-015-0071-z

**Published:** 2015-04-10

**Authors:** Zachery T Lewis, Sarah M Totten, Jennifer T Smilowitz, Mina Popovic, Evan Parker, Danielle G Lemay, Maxwell L Van Tassell, Michael J Miller, Yong-Su Jin, J Bruce German, Carlito B Lebrilla, David A Mills

**Affiliations:** Department of Food Science and Technology, UC Davis, 1 Shields Avenue, Davis, CA 95616 USA; Department of Chemistry, UC Davis, 1 Shields Avenue, Davis, CA 95616 USA; Department of Viticulture and Enology, UC Davis, 1 Shields Avenue, Davis, CA 95616 USA; Foods For Health Institute, UC Davis, 1 Peter J Shields Avenue, Davis, CA 95616 USA; Department of Life Sciences, PhD School in Science and Technologies for Health Products, University of Modena and Reggio Emilia, Via Università, 4, Modena, MO 41100 Italy; Genome Center, UC Davis, 1 Shields Avenue, Davis, CA 95616 USA; Department of Food Science and Human Nutrition, University Illinois at Urbana-Champaign, S. Goodwin Ave., Urbana, IL 61801 USA

**Keywords:** Bifidobacteria, Secretor, Infant, Breastfeeding, FUT2, Marker gene sequencing, Human milk oligosaccharides, Short-chain fatty acids

## Abstract

**Background:**

Individuals with inactive alleles of the fucosyltransferase 2 gene (FUT2; termed the ‘secretor’ gene) are common in many populations. Some members of the genus *Bifidobacterium*, common infant gut commensals, are known to consume 2′-fucosylated glycans found in the breast milk of secretor mothers. We investigated the effects of maternal secretor status on the developing infant microbiota with a special emphasis on bifidobacterial species abundance.

**Results:**

On average, bifidobacteria were established earlier and more often in infants fed by secretor mothers than in infants fed by non-secretor mothers. In secretor-fed infants, the relative abundance of the *Bifidobacterium longum* group was most strongly correlated with high percentages of the order *Bifidobacteriale*s. Conversely, in non-secretor-fed infants, *Bifidobacterium breve* was positively correlated with *Bifidobacteriale*s, while the *B. longum* group was negatively correlated. A higher percentage of bifidobacteria isolated from secretor-fed infants consumed 2′-fucosyllactose. Infant feces with high levels of bifidobacteria had lower milk oligosaccharide levels in the feces and higher amounts of lactate. Furthermore, feces containing different bifidobacterial species possessed differing amounts of oligosaccharides, suggesting differential consumption *in situ*.

**Conclusions:**

Infants fed by non-secretor mothers are delayed in the establishment of a bifidobacteria-laden microbiota. This delay may be due to difficulties in the infant acquiring a species of bifidobacteria able to consume the specific milk oligosaccharides delivered by the mother. This work provides mechanistic insight into how milk glycans enrich specific beneficial bacterial populations in infants and reveals clues for enhancing enrichment of bifidobacterial populations in at risk populations - such as premature infants.

**Electronic supplementary material:**

The online version of this article (doi:10.1186/s40168-015-0071-z) contains supplementary material, which is available to authorized users.

## Background

The establishment of the intestinal microbiota after birth is an important event in the life of a newborn [1]. *Bifidobacterium* species are among the early colonizers of breastfed infants [2], with evidence that they are uniquely beneficial to the newborn infant in various ways [3-7]. Non-digestible sugars in breast milk known as human milk oligosaccharides (HMOs) are protective to infants [8] and function as a prebiotic in the establishment of bifidobacteria. Select members of the genus *Bifidobacterium* commonly found in breastfed infants are able to utilize HMOs as carbon sources, including fucosylated oligosaccharides [9-13]. The relationship between mothers, infants, and bifidobacterial species appears to have co-evolved over mammalian history [14], perhaps to aid the infant in avoiding infection. Bifidobacteria have also been shown to reduce inflammation and gut permeability [7,15-17]. A recent analysis of breastfed infants in Bangladesh revealed that higher bifidobacterial populations correlate with improved responses to both oral and parenteral vaccines early in infancy [5]. Bifidobacteria are not alone in their ability to consume HMOs, as members of the genus *Bacteroides* are known to consume some types of HMOs [18]. These two groups are both involved in the production of short-chain fatty acids and lactate, which alter the pH of the environment, modulate the microbiota, and have other systemic properties [19].

HMOs can be bound to other compounds in milk as glycoconjugates, which may play a similar role to free HMOs [20]. Together, free HMOs and their related glycoconjugates have been referred to as human milk glycans (HMGs) [6]. Among the genes that build HMGs in the mammary gland is the fucosyltransferase 2 (FUT2) gene, which catalyzes the transfer of fucose residues by an α1,2-linkage to glycans found in human milk. Known as the ‘secretor’ gene because of its role in the expression of ABO blood types in various secreted body fluids (tears, saliva, breast milk, and so on), this gene has well-known mutations that inactivate transferase activity which occur in most populations across the world, including in about 20% of the population of the United States [21]. FUT2 seems to be under balancing selection [22,23], as there are both advantages and disadvantages to possessing an active copy of the gene. For example, non-secretors are resistant to rotavirus [24], norovirus [25], and *Helicobacter pylori* [26] infections, while secretors have lower risk in developing type 1 diabetes [27] and Crohn’s disease [28]. Breastfeeding mothers who are secretors also confer resistance to diarrheal disease on their children [29]. Morrow *et al.* found that there were differences in survival between premature infants of differing secretor statuses [30], although how much the mother’s genotype plays into this outcome is unknown. The amount of fucosylation in breast milk is also known to change over the course of lactation [31], which may affect the protection conferred to an infant over time.

There are phenotypic differences in the milk glycans from secretors and non-secretors [32] and in their ability to deflect pathogen binding to the epithelium [33]. HMOs containing α1,2-fucosyl linkages have been shown to promote the growth of bifidobacteria due to prebiotic action [34]. *Bifidobacterium longum* subsp. *infantis* and *Bifidobacterium bifidum* possess glycosyl hydrolase family 95 (GH95) fucosidases that act on 2′-fucosylated HMOs [12,35]. In other bifidobacteria such as *Bifidobacterium breve*, GH29 fucosidases enable the consumption of 2′-fucosylated HMOs [36]. Regardless, there is little known about how actual breast milk from mothers of different secretor statuses affects the resulting gut community of a breastfed infant. This study examines the differences in infant gut microbial populations that arise from these compositional differences in HMGs.

## Results

### Maternal secretor status

A subset of 44 infant/mother dyads from the existing UC Davis Foods For Health Institute Lactation Study were selected for an analysis of the effects of a mother’s secretor status on her infant’s gut microbiota over four time points ranging from 7 to 120 days of life. To determine each mother’s secretor status, several specific 2′-fucosylated HMO ‘markers’ were quantitated in the earliest milk available from each mother (Additional file 1: Table S1). Of the 107 milk samples, 35 were found to be from 12 non-secretor donors (33%) and 72 were from 32 secretor donors (67%). Aside from the levels of secretor status marker oligosaccharides, milk determined to be from non-secretor mothers showed other significant differences in glycan composition when compared to secretor milk over all four time points (Table 1). Although the total oligosaccharide abundance and the relative amount of total (including 2′ and 3′) fucosylation were comparable among averaged data from the two phenotypes, the relative abundances of non-fucosylated neutral and sialylated structures differed. In non-secretors, the average amount of sialylation was 23.4% ± 5.7%, which is significantly higher than the amounts of sialylation found in secretors, which averaged 18.2% ± 4.8% (*p* < 0.0001). Conversely, non-secretor milk had lower relative amounts of non-fucosylated neutral structures than secretor milk, with 21.3% ± 8.8% and 25.4% ± 7.3%, respectively (*p* = 0.023).

**Table 1:** **Secretor phenotype characteristics**

**Measurement**	**Non-secretor**	**Secretor**	**2-tailed** ***t*** **-test** ***p*** **value**
Sample size (*N*)	35	72	N/A
Total HMO signal (ion counts ± SD)	5.11e08 ± 1.52e08	5.61e08 ± 1.46e08	0.112
Fucosylation (ion counts ± SD)	3.55e08 ± 1.14e08	3.67e08 ± 9.50e07	0.569
% fucosylation	68.3 ± 7.8	65.8 ± 7.5	0.119
Sialylation (ion counts ± SD)	1.25e08 ± 5.19e07	1.04e08 ± 4.28e07	0.044
% sialylation	23.4 ± 5.7	18.2 ± 4.8	<0.0001
Non-fucosylated neutral (ion counts ± SD)	1.02e08 ± 4.17e07	1.44e08 ± 5.52e07	<0.0001
% non-fucosylated neutral	21.3 ± 8.8	25.4 ± 7.3	0.012

Means, standard deviations, and *t*-test values of each HMO (human milk oligosaccharide) class by secretor phenotype.

To rule out some potential external factors that could confound a microbiota comparison between the two secretor phenotypes, maternal and infant demographics and clinical characteristics were compared between the two groups. No obvious differences between secretor and non-secretor groups were found (Tables 2 and 3). All infants consumed breast milk throughout the study duration; however, based on parental reports, some infants occasionally consumed limited amounts of supplemental infant formula and/or solid foods (see Additional file 2: Table S2 for details). Additional file 2: Table S2 also indicates from which infants samples were available for each of the four time points.

**Table 2:** **Reported maternal demographics and characteristics**

**Maternal characteristic**	**Secretor (** ***n*** **= 32)**	**Non-secretor (** ***n*** **= 12)**
Maternal education		
High school	1	0
Bachelor’s degree	11	1
Master’s degree	12	7
PhD or equivalent	8	4
Maternal ethnicity		
Caucasian	29	10
Asian	1	1
Hispanic	2	1
Secretor genotype^a^		
SS	6	0
Ss	25	0
ss	0	12
Maternal blood type (ABO)		
Type O	15	6
Type A	15	5
Type B	1	1
Type AB	1	0
Maternal Rh factor		
Positive	29	9
Negative	3	3
Parity		
Primiparous	24	8
Multiparous	8	4
Birth mode		
Vaginal	28	11
C-section	4	1
Infant gender		
Female	17	7
Male	15	5

Values are frequencies per total sample size, *n* = 44. ^a^Genotyping data missing for one subject phenotypically described as a secretor.

**Table 3:** **Maternal and infant clinical characteristics**

**Characteristic**	**Secretor (** ***n*** **= 32)**	**Non-secretor (** ***n*** **= 12)**
Maternal age, years	31.1 ± 4.1 (24.0 to 45.0)	32.8 ± 3.5 (27.0 to 39.0)
Maternal BMI, pre-pregnancy^a^, kg/m^2^	24.3 ± 4.7 (18.0 to 37.0)^b^	22.9 ± 3.9 (18.9 to 33.7)
Infant gestational age at birth^c^, week	39.7 ± 1.3 (37.0 to 42.1)	39.8 ± 1.2 (37.0 to 41.5)
Infant birth weight^c^, g	3,460 ± 399 (2,890 to 4,390)	3,530 ± 455 (2,660 to 4,370)
Infant birth length^c^, cm	51.6 ± 2.0 (47.0 to 57.2)	51.3 ± 1.8 (48.9 to 55.9)
Breast milk consumption post birth^c^, hours	3.1 ± 5.6 (0.3 to 24.0)	2.8 ± 5.8 (0.2 to 21.0)

Values, mean ± SD (range). ^a^Self-reported data for the time preceding pregnancy, *n* = 44. ^b^ *n* = 30. ^c^Self-reported, *n* = 44.

To validate the phenotypic designation (secretor or non-secretor) assigned to each mother, genotypic information about secretor status was also generated for each mother. Mothers determined to be homozygote non-secretors by genotype were, in all cases, also determined to be non-secretors in phenotype as described above. In two cases (mothers 1036 and 1041), the genotypic data showed either homozygote secretor or heterozygote (respectively), but the phenotype indicated non-secretor. In all other cases, the homozygotic and heterozygotic secretors were determined to have a secretor phenotype. In the two aberrant cases, later time points revealed secretor levels of 2′-fucosylation in these two mothers, suggesting that secretor phenotype might change over the course of lactation in some mothers.

### Fecal bifidobacterial levels

**Figure 1:**
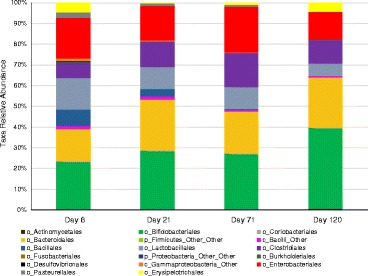
**Average fecal bacterial populations in the infant cohort over time.** Taken from order-level or higher classification levels of the marker gene sequencing data.

To investigate if the grouping by mother’s secretor status produced differences in the gut microbiota of the infant, we used 16S rRNA gene amplicon sequencing to probe the fecal microbiota of the infants. In general, the most common bacterial groups found in all the infants were *Bifidobacteriales*, *Lactobacillales* (mostly *Streptococcus*), *Bacteroides*, *Enterobacteriaceae*, and *Clostridiaceae*. As shown in Figure 1, there is a general trend towards increasing amounts of *Bacteroidales* and *Bifidobacteriales* and decreasing amounts of *Lactobacillales* (mostly *Streptococcus*) and *Enterobacteriales* over time. Differences were seen between the five C-section-born infants and the 39 vaginally born infants, with C-section infants having much lower levels of bifidobacteria and *Bacteroides*, although the number of C-section infants was too low to draw any robust conclusions (Additional file 3: Figure S1).

**Figure 2:**
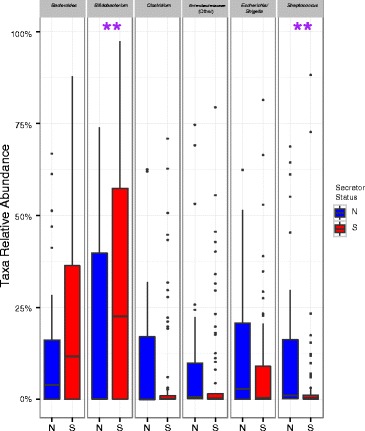
**Comparison of relative levels of gut microbiota in secretor**-**fed infants and non**-**secretor-fed infants.** Asterisks indicate significant differences (*p* < 0.05) in the relative levels of various gut microbes using a Wilcoxon rank sum test. The color boxplots show the quartiles above and below the median; the dark line near the center of the box denotes the median. The whiskers extend to the first and fourth quartiles, and the black dots show outliers. N = non-secretor, S = secretor.

**Figure 3:**
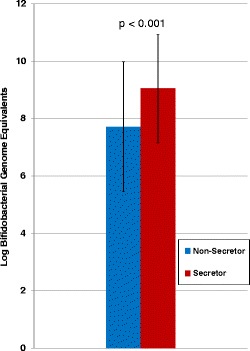
**Average absolute levels of bifidobacteria in secretor** ***versus*** **non**-**secretor**-**fed infants (all samples of each secretor status averaged together).** The one-tailed type three *t*-test *p* value was <0.001.

**Figure 4:**
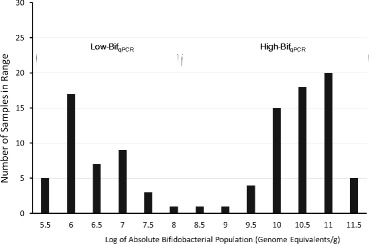
**Histogram of absolute bifidobacterial populations.** Bimodal distribution of results from bifidobacterial qPCR showing the lack of intermediate levels.

**Figure 5:**
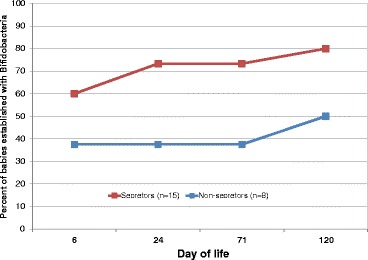
**Percentage of infants with high bifidobacteria over time.** Based on when each qualifying infant crossed the cutoff point of 10^8.5^ bifidobacterial genome equivalents/gram feces. Infants qualified for this analysis by having the appropriate time points available to know when they are first established with bifidobacteria; for example, if the day 6 sample is missing, it is impossible to know if the infant was established at that time or not, and thus, that infant was excluded from this analysis.

When grouped by the mothers’ secretor status, differences were seen in the aggregated infants’ gut microbiota (Figure 2). Specifically, secretor-fed infants generally had higher relative amounts of *Bifidobacterium* (*p* < 0.05) and *Bacteroides* and lower levels of enterobacteria, clostridia, and streptococci (*p* < 0.05). To verify the higher levels of bifidobacteria found in secretor-fed infants, we probed the samples with bifidobacterial-specific quantitative PCR (qPCR). The aggregated secretor-fed infant samples were, on average, found to have significantly higher absolute levels of bifidobacteria (10^9.0^/g feces *versus* 10^7.7^/g feces, *p* < 0.001) (Figure 3). The distribution of the qPCR data was bimodal, with a large group of samples with low levels of bifidobacteria (<10^7.6^/g feces, average 10^6.1^) and a separate group with high amounts of bifidobacteria (>10^8.9^/g feces, average 10^10.3^), with a striking lack of values falling in between the two ranges (Figure 4). These two groupings of samples were labeled ‘Low-Bif_qPCR_’ and ‘High-Bif_qPCR_’ samples, respectively. Much of the bifidobacterial abundance difference between the two secretor status milk phenotypes appears to come from variation in the time at which each infant transitions from possessing a Low-Bif_qPCR_’ gut community to possessing a High-Bif_qPCR_ gut community. Bifidobacteria were found to be established (High-Bif_qPCR_) earlier in secretor-fed infants (60% of infants *versus* 37.5% at day 6) and more often (80% *versus* 50% by day 120) (Figure 5).

**Figure 6:**
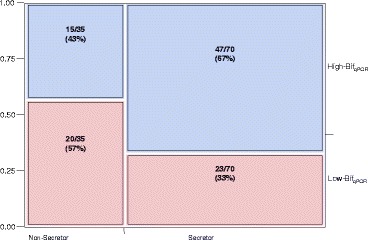
**Contingency plot of secretor status by bifidobacterial content.** Pearson chi-square test was significant (*p* = 0.0171), indicating that mother’s secretor status and infant bifidobacteria levels are dependent variables. A Fisher’s exact test yielded *p* = 0.015.

To examine the dependence of bifidobacterial abundance in stool on secretor status phenotype in milk, a contingency analysis was performed on the 105 available matching milk and stool pairs (Figure 6). Of 35 milks from non-secretors, 20 of the matched infant stool samples were Low-Bif_qPCR_ (57.1%). Of the 70 milks from secretor women, 23 of the matched infant stool samples were Low-Bif_qPCR_ (32.9%). A Pearson chi-square test was significant (*p* = 0.0171), indicating that mother’s secretor status and infant bifidobacteria levels are dependent variables. A Fisher’s exact test yielded *p* = 0.015, suggesting the probability that bifidobacteria levels will be High-Bif_qPCR_ is greater for infants who are receiving milk from secretor mothers.

**Figure 7:**
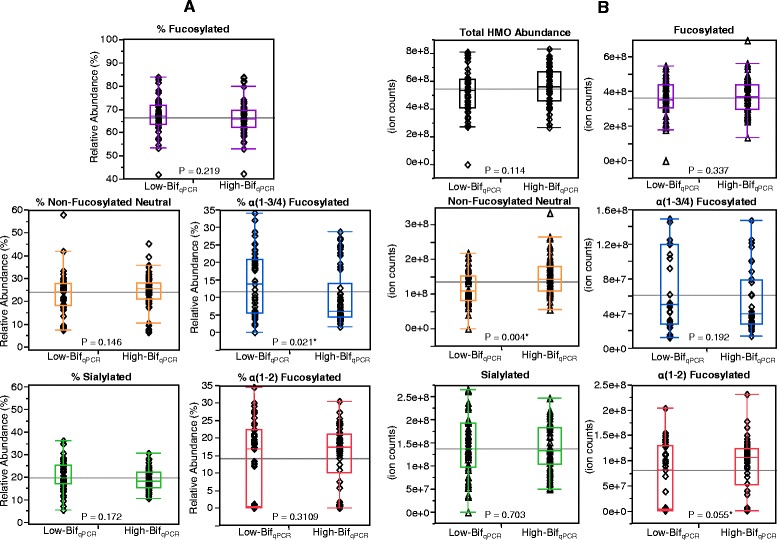
**Differences in milk HMOs fed to infants that were High**-**Bif**_**qPCR**_ **or Low**-**Bif**_**qPCR**_ **in the corresponding feces. (A)** shows relative abundance and **(B)** shows absolute abundance. The *p* values are from a two-tailed unpaired *t*-test. * = Significant at 95% confidence level. HMO = human milk oligosaccharide.

We then tested whether differences in the types and amounts of oligosaccharides present in the milk other than 2′-fucosylation leads to differences in the amount of bacteria present. Figure 7 shows that stool samples with Low-Bif_qPCR_ counts (*N* = 43) and those with High-Bif_qPCR_ counts (*N* = 62) were from infants that received matching milk samples of mostly comparable glycan composition. On average the milk received by Low-Bif_qPCR_ infants was significantly higher in α(1-3/4)-fucosylated oligosaccharides than milk received by the High-Bif_qPCR_ infants (14.2% ± 9.2% *versus* 10.1 ± 7.9%, respectively; *p* = 0.021), but this may be mostly due to the trade-off between 2′- and 3′-fucosylation related to secretor status, which was shown above to correlate with an increase in bifidobacterial abundance. In absolute ion counts per microliter of milk, the High-Bif_qPCR_ group of infants received milk marginally, but significantly higher in non-fucosylated neutral glycans (1.5e08 ± 5.3e07 counts *versus* 1.2e08 ± 4.7e07 counts, *p* = 0.004) and α(1-2)-fucosylated glycans (9.1e07 ± 5.3e07 counts *versus* 6.8e07 ± 6.4e07 counts, *p* = 0.055 two-tailed *t*-test, *p* = 0.028 one-tailed *t*-test).

### Fecal glycoprofiles

**Figure 8:**
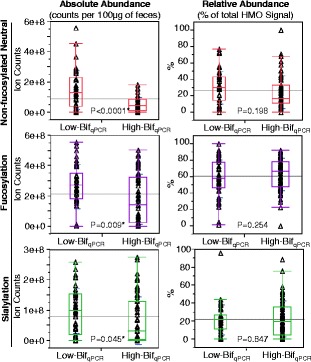
**Differences in fecal HMOs between samples that were either High**-**Bif**_**qPCR**_ **or Low**-**Bif**_**qPCR**_ **.** The *p* values are from a two-tailed unpaired *t*-test. * = Significant at 95% confidence level. HMO = human milk oligosaccharide.

To test whether bifidobacteria in general were a driver of oligosaccharide consumption in the infant gut, we measured types and amounts of oligosaccharides in the feces as a proxy for (lack of) consumption. Of 107 stool samples, glycans were detected in 103, of which bifidobacterial abundance was also measured in 102 samples. Figure 8 shows a breakdown of how fecal glycoprofiles differed between samples that were High-Bif_qPCR_ and Low-Bif_qPCR_. The absolute abundance of fecal glycans (residual milk glycans present in infant stool) were significantly higher in Low-Bif_qPCR_ stool samples (*N* = 42) than in High-Bif_qPCR_ (*N* = 60). Significantly higher amounts of non-fucosylated neutral (*p* < 0.0001), fucosylated (*p* = 0.009), and sialylated species (*p* = 0.045) were left behind in the Low-Bif_qPCR_ fecal samples, as determined in ion counts per 100 μg of stool. The two groups showed no significant differences in glycan composition in terms of relative abundance (percent of total HMO signal) between the three glycan types.

### Fecal bifidobacterial isolates

**Figure 9:**
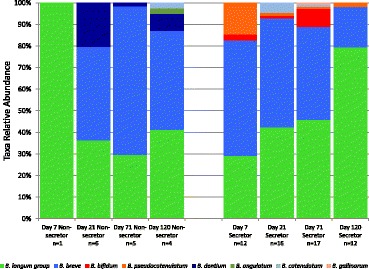
**Bifidobacterial isolates obtained from fecal samples.** Data from 382 isolates from 38 babies across 73 samples. Eight species of bifidobacteria were detected. The *B. longum* group and *B. breve* were the most commonly detected. Other species detected include *B. pseudocatenulatum*, *B. catelanum*, *B. gallinarum*, *B. bifidum*, *B. dentium*, and *B. angulatum*. Non = non-secretor-fed, Sec = secretor-fed, and *n* = number of samples from which isolates were obtained. The ‘n’ denotes the number of samples represented in each bar, not the number of isolates.

**Figure 10:**
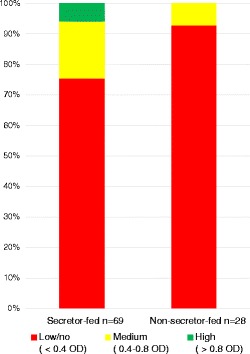
**Bifidobacterial isolates growth on 2**′-**fucosyllactose.** The OD achieved by each strain during growth on 2′-fucosyllactose (2FL) was compared with the OD obtained in the absence of sugar source as a negative control and lactose as a positive control. This difference in OD (ΔOD) was used as a parameter to evaluate the strain’s ability to grow on the 2FL.

Noting the previous differences in fecal glycoprofiles, we hypothesized that secretor mothers would enrich their infants in bifidobacteria that are able to consume 2′-fucosylated HMOs. To test whether there were functional differences in the ability of bifidobacteria from infants with mothers of differing secretor statuses to consume a secretor-type oligosaccharide (2′-fucosyllactose), we obtained over 400 isolates from the 107 infant fecal samples in this study for the purpose of growing them *in vitro* with only 2′-fucosyllactose as a carbon source. Of these isolates, 382 were identified by the matrix-assisted laser desorption ionization (MALDI) Biotyper (Bruker, Fremont, CA, USA) as belonging to the genus *Bifidobacterium*. Bifidobacteria were successfully isolated from 73 of the 107 samples. Figure 9 shows a breakdown of these isolates by species and mother’s secretor type over each of the four time points. The most commonly isolated species were *B. breve* and members of the *B. longum* group. As MALDI may not reliably distinguish between members of this group, we do not include subspecies designations in the description of the isolates. Notably, the *B. longum* group increased in proportional representation over time in the secretor-fed, but not in the non-secretor-fed infants. This data may however be skewed by isolation bias. Of the 382 bifidobacteria isolates, 97 were chosen as a subset of ‘unique’ isolates to study further. The ‘unique’ subset included only one isolate of each bifidobacterial species obtained from each sample, selected at random from among isolates of the same species. Multiple isolates of the same species from the same fecal sample are likely from a clonal population in the infant gut, thus our designation of these isolates as the ‘unique’ subset [37]. This subsetting was necessary due to limitations in the amount of 2′-fucosyllactose growth substrate available. Each unique isolate was grown on 2′-fucosyllactose (2FL) to test its ability to consume this prototypical secretor-type sugar. Using the cutoff maximum optical densities (ODs) shown in the example growth curves in Additional file 4: Figure S2, we classified each isolate as a high, medium, or low grower. Notably, a higher percentage of isolates from secretor-fed infants grew to medium and high ODs than from non-secretor-fed infants (Figure 10). In addition, of the two isolates obtained from non-secretor-fed infants that could grow on 2FL, one was a *Bifidobacterium dentium* strain, a species adapted to oral niches [38,39].

### Fecal bifidobacterial (sub)species profiles

**Figure 11:**
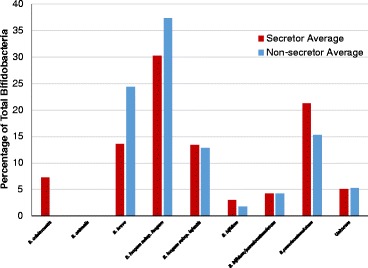
**Bifidobacterial species content**: **secretor** ***versus*** **non**-**secretor.** Based off of Bif-TRFLP (bifidobacteria-specific terminal restriction fragment length polymorphism) and BLIR (*Bifidobacterium longum*/*infantis* ratio) data. Some peaks in the electropherogram from Bif-TRFLP could correspond to either *B. bifidum* or *B. pseudocatenulatum* and are listed as such in their own category.

**Figure 12:**
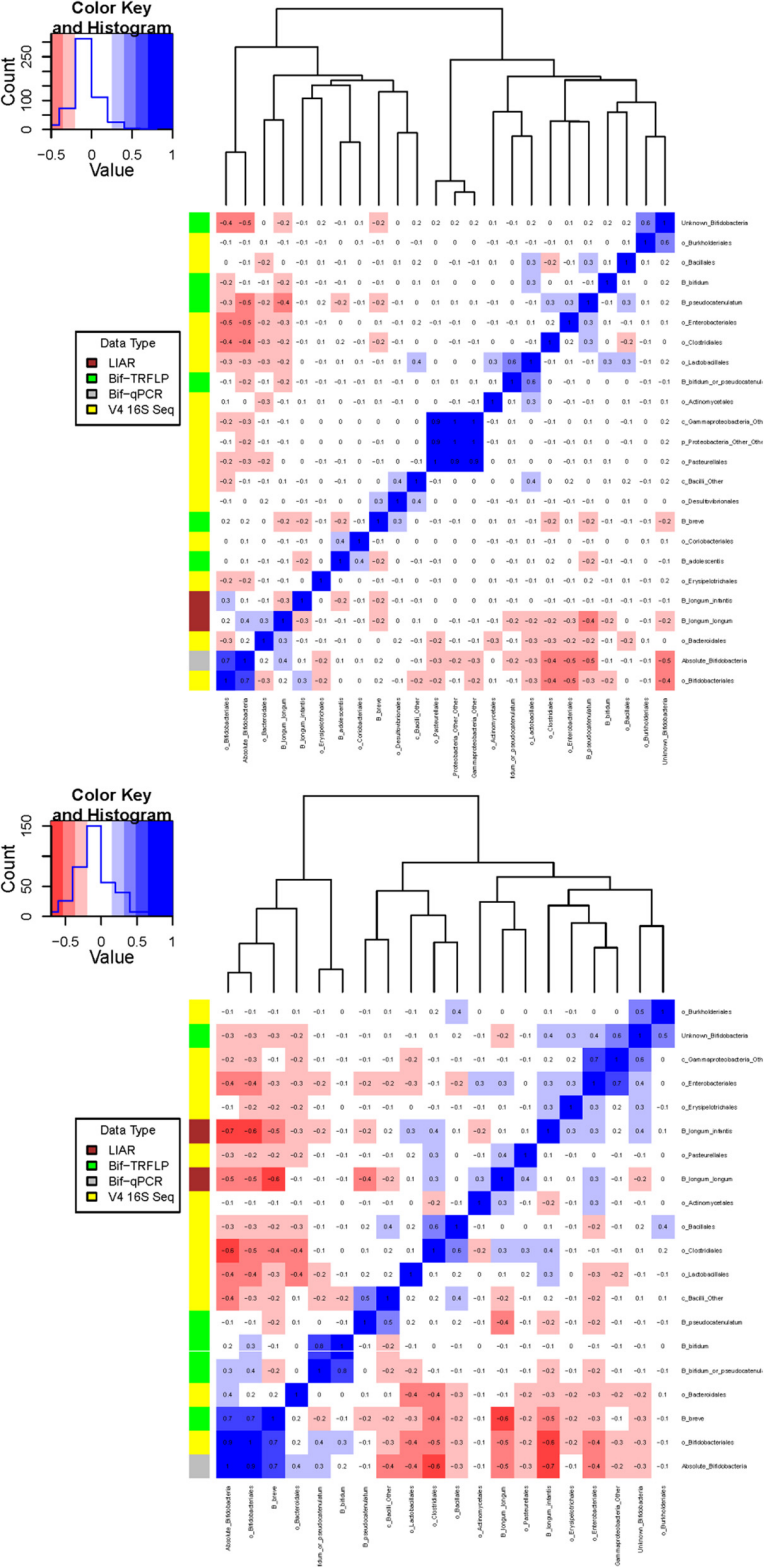
**Pearson correlation matrices.** Correlating the results of Bif-TRFLP (bifidobacteria-specific terminal restriction fragment length polymorphism), qPCR, V4 16S amplicon sequencing, and BLIR (*Bifidobacterium longum*/*infantis* ratio) in non-secretor-fed (bottom) and secretor-fed (top) infants. The number in each box is the Pearson correlation coefficient. The colored bar on the left side of the matrix indicates what type of data the row is. Negative correlations are colored in shades of red and positive correlations are shown in shades of blue.

As different species and subspecies of bifidobacteria have differences in their glycan consumption capabilities, we investigated whether secretor- and non-secretor-fed infants differed in their bifidobacterial content at a taxonomic resolution higher than achievable by our marker gene sequencing method (with current read lengths). At first glance, it appeared as if there were no species-level differences in the bifidobacterial populations of secretor-fed and non-secretor-fed infants (Figure 11). However, a difference was noted between secretor-fed infants and non-secretor-fed infants in which bifidobacterial species were present in high relative abundances when compared to the microbiota as a whole. Specifically, there was a difference in the correlation of the relative abundance of various bifidobacterial species (from the bifidobacteria-terminal restriction fragment length polymorphism (Bif-TRFLP) data) with absolute (qPCR) and relative (amplicon sequencing) bifidobacterial abundance (Figure 12, top and bottom). Both the *B. longum* group and *B. breve* are positively correlated with bifidobacterial abundance in secretor-fed infants, while only *B. breve* holds that distinction in non-secretor-fed infants (*B. bifidum* is shown as correlated, but is only present in one non-secretor-fed sample for which marker gene sequencing data is present, and at a level of only 3% of total bifidobacteria). Interestingly, the *B. longum* group is strongly anti-correlated with bifidobacterial abundance in non-secretor-fed infants. *B. longum* subsp. *infantis* was not found in a single high-bifidobacteria sample from non-secretor-fed infants (data not shown). Phrased differently, distinct sets of bifidobacterial species seem to be able to dominate the community in infants fed by mothers of different secretor statuses.

**Figure 13:**
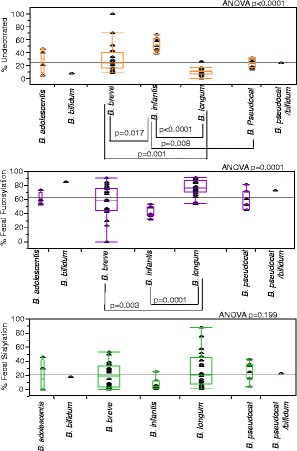
**Differences in the amount of fecal HMOs in infants dominated by different species of bifidobacteria.** Only samples that were High-Bif_qPCR_ were included in this figure.

To test whether the presence of different bifidobacterial species led to differences in oligosaccharide content of the feces (and thus putative oligosaccharide consumption), we compared the fecal glycome between samples possessing different dominant species of bifidobacteria (Figure 13). Of the 60 stool samples with high-bifidobacterial abundances, the species identified were *Bifidobacterium adolescentis* (*N* = 4), *B. bifidum* (*N* = 1), *B. breve* (*N* = 20), *B. longum* subsp. *infantis* (*N* = 7), *B. longum* subsp. *longum* (*N* = 20), *Bifidobacterium pseudocatenulatum* (*N* = 7), and the ambiguous *B. pseudocatenulatum*/*bifidum* designation sometimes arising in the course of Bif-TRFLP analysis (*N* = 1). Bifidobacterial species identified in stools with low bifidobacteria abundance were *B. bifidum* (*N* = 1), *B. breve* (*N* = 1), *B. longum* subsp. *infantis* (*N* = 1), *B. longum* subsp. *longum* (*N* = 10), *B. pseudocatenulatum* (*N* = 22), and *B. pseudocatenulatum*/*bifidum* (*N* = 1). In six samples, *B. longum* subsp. *infantis* and *B. longum* subsp. *longum* were of roughly equal abundance and, thus, were designated more generally as ‘*B. longum* group’. Species groups with *N* < 4 were omitted from statistical analysis. As expected, glycan presence was not significantly different between samples with different major bifidobacterial species in Low-Bif_qPCR_ stool, as bifidobacteria were not likely the major glycan consumer in samples where they were not abundant (data not shown). In High-Bif_qPCR_ stool, there were significant differences in the percentage of fucosylated species (ANOVA *p* = 0.0001) and the percentage of non-fucosylated neutral species (ANOVA *p* < 0.0001). By a pairwise comparison, samples high in *B. longum* subsp. *infantis* were shown to have a higher percentage of non-fucosylated neutral oligosaccharides left behind in the feces. Of the oligosaccharides not ostensibly consumed by *B. longum* subsp. *infantis*, 52.3% ± 5.8% were non-fucosylated neutral species on average. The relative abundance of non-fucosylated neutral species was considerably lower in samples high in all the other oligosaccharide types, all with averages ≤30%. The percentage of fucosylated oligosaccharides among the residual fecal glycans was highest in infants with *B. longum* subsp. *longum* as their dominant species of bifidobacteria, with an average of 77.3% ± 3.7%. This value was significantly higher than the fucosylated oligosaccharide percentages leftover in samples dominated by *B. breve* (57.3% ± 3.7%, *p* = 0.003) and *B. longum* subsp. *infantis* (42.7% ± 6.3%, *p* = 0.0001).

### Broader infant bacterial communities

As we had observed the impact of mother’s secretor status on the amount and type of bifidobacteria in the infant’s feces, we wished to investigate the impact of secretor status on the rest of the infant’s gut community structures. We first classified infant gut communities in a less-supervised manner, which would independently capture important differences in community structures while requiring fewer *a priori* choices on our part. Using the QIIME 1.8.0 implementation of the BIO-ENV function of the ‘vegan’ R package (‘BEST’), a subset of the most abundant bacterial species were tested for their impact on community UniFrac distances [40]. Results indicated that genera *Bifidobacterium* and *Bacteroides* were the top two contributors to differences among the given inputs (rho statistic = 0.586 when only those two factors are considered, see Additional file 5: Table S3). A principle coordinate analysis of the marker gene sequencing results revealed three main clusters of samples, (Additional file 6: Figure S3, middle plot) with one outlier. The three clusters were respectively distinguished by high bifidobacterial content (BI), high *Bacteroides* (BA), and high levels of a number of other taxa including streptococci, enterobacteria, and clostridial species (OT) (see Additional file 6: Figures S3, Additional file 7: Figure S4, Additional file 8: Figure S5, Additional file 9: Figure S6). The one outlier sample that fell into its own category (high enterococci) was from a C-section infant (Additional file 6: Figure S3). Infants often moved between groups over time, and by day 120, few infants remained in the OT group (Additional file 6: Figure S3). Notably, a higher abundance of non-secretor-fed infants fell into the OT area of the plot (Additional file 6: Figure S3). The infant stools were thus divided into groups of *Bacteroides* (BA_PCoA_, *N* = 24), bifidobacteria (BI_PCoA_, *N* = 38), or OT_PCoA_ (*N* = 39) dominated samples, based on the PCoA groupings described previously. ANOSIM was used to test the explanatory power of this grouping, with positive results (R statistic = 0.7887, *p* = 0.001 after 999 permutations, see Additional file 10: Table S4).

**Figure 14:**
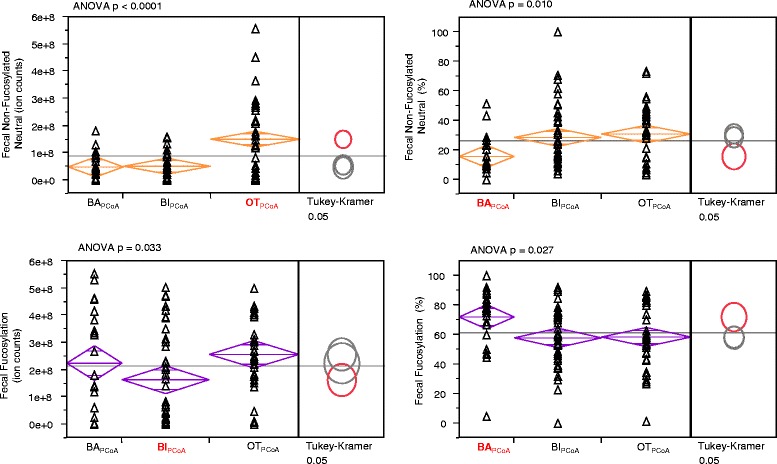
**Differences in the amount of fecal HMOs in infants of each PCoA group.** Differences in the amount of each HMO class remaining in the feces of samples that fell into each of the three main PCoA categories BA_PCoA_ = *Bacteroides*, BI_PCoA_ = *Bifidobacterium*, OT_PCoA_ = other taxa.

To investigate the impacts of having a microbiota dominated by taxa other than bifidobacterial species, we compared fecal glycomes across these three groups using ANOVA (Figure 14). There were significant differences in total oligosaccharide abundance (*p* = 0.0003) among the three groups, as well as differences in the absolute and relative abundances of fucosylated (*p* = 0.033 absolute, *p* = 0.027 relative) and non-fucosylated neutral (*p* < 0.0001 absolute, *p* = 0.010 relative) oligosaccharide types. The OT_PCoA_ group differed the most from the BI_PCoA_ and BA_PCoA_ groups in terms of absolute abundance of non-fucosylated neutral oligosaccharides (Tukey-Kramer honestly significant difference (HSD) *p* < 0.0001 and *p* = 0.0002, respectively), having significantly higher oligosaccharide amounts in the stool, with an average of 1.51e08 ± 1.45e07 counts in the OT_PCoA_ group, *versus* 5.20e07 ± 1.47e07 counts and 5.12e07 ± 1.90e07 counts in the BI_PCoA_ and BA_PCoA_ groups, respectively. The *relative* abundance of non-fucosylated neutral species differed most in the BA_PCoA_ group (accounting for 16.0% ± 3.9% of the total), being significantly lower than those in the BI_PCoA_ group (28.5% ± 3.0%, *p* = 0.035) and OT_PCoA_ group (30.8% ± 2.9%, *p* = 0.009). Additionally, the BA_PCoA_ group also had a higher percentage of fucosylated oligosaccharides than the other two groups, with 71.3% ± 4.2% fucosylation, *versus* 57.9% ± 3.3% in the BI_PCoA_ group (*p* = 0.035) and 58.44% ± 3.2% in the OT_PCoA_ group (*p* = 0.044). The absolute abundance of fucosylation in the OT_PCoA_ group (2.57e08 ± 2.52e07 ion counts) was significantly higher than that of the BI_PCoA_ group (1.64e08 ± 2.56e07 ion counts, *p* = 0.029). Absolute and relative amounts of sialylation were similar across all three groups.

### Fecal lactate

**Figure 15:**
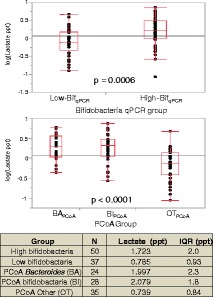
**Lactate concentrations by bifidobacteria qPCR group or PCoA group.** qPCR group (top) or PCoA group (bottom). The *p* values are from a two-tailed Student’s *t*-test. BA_PCoA_ = *Bacteroides*, BI_PCoA_ = *Bifidobacterium*, OT_PCoA_ = other taxa.

Bifidobacteria produce two main metabolic end products: lactate and acetate [41]. As a measure of metabolic output from oligosaccharide consumption, we investigated whether the concentration of lactate varied with microbiota composition. Lactate was chosen due to its lower volatility than acetate. Figure 15 shows a summary of the differences in fecal lactate that correlate with microbiota differences. The results are from a subset of the samples, as only 87 samples had sufficient sample quantity for the analysis (updated group sizes are included with the tabulated result). Lactate concentration did not appear to be normally distributed nor did the Bartlett test indicate that most groupings were homoscedastic. Accordingly, log transformations were performed prior to statistical analysis, and the results are tabulated as the median in parts per thousand (ppt) by mass along with the interquartile range.

When comparing groups based off of the bifidobacterial-specific qPCR data (high/low), the High-Bif_qPCR_ group was found to have higher levels of lactate (*p* = 0.0006). In the groups based off of PCoA clustering (BA_PCoA_, BI_PCoA_, OT_PCoA_), lactate was found to have significant differences by ANOVA. Pairwise comparison of the lactate results found no significant differences between BI_PCoA_ and BA_PCoA_ groups, but found that the OT_PCoA_ group was lower than both the BI_PCoA_ group and the BA_PCoA_ group with *p* < 0.0001 in each case.

## Discussion

While breastfeeding is overwhelmingly recommended as the best source of nutrition for a newborn, it is clear that not all breast milk is the same. Maternal diet, gestational age of the infant, and lactation stages have been known to influence the lipid and protein content of milk [42,43]. Conversely, term milk glycan compositions appear relatively stable throughout lactation, perhaps highlighting their importance to the developing infant [44]. Decreasing milk glycan concentrations over the course of lactation have been observed, for which the increased volume consumed by infants at later stages of nursing may compensate [45]. Milk glycan composition from mothers who deliver prematurely was recently shown to be more variable than mothers delivering term infants [46]. Milk composition is also affected by the mother’s glycosyltransferase genotype [47]. In this study, the milk of secretors and non-secretors had modest differences besides the amount of 2′-fucosylation. Secretors had higher absolute amounts of sialylated sugars and higher relative amounts of undecorated sugars. Non-secretors had higher relative amounts of sialylated sugars. These factors may also play a part in the shaping of the secretor-fed infants’ microbiota. The potentially confounding factor of the infant’s own glycosylation system, the timeline and levels of its expression, and its potential influences on the microbiota were not explored in this study and remain to be elucidated.

It is well established that differences in gut glycan content affect the gut microbiota. Secretor status has been shown to affect both the gut microbiota and metabolite profiles in adults [48,49]. Secretor status also specifically affects the species composition and absolute abundance of gut bifidobacterial populations in adults, with secretors having higher bifidobacterial abundance [28,50]. Numerous studies have also associated intake of milk glycans with the initial colonization of the infant gastrointestinal tract [34,51,52].

Our data provide insight into what types of milk inputs are most likely to lead to a high-bifidobacteria microbiota in the context of our cohort (healthy infants in a developed nation). The strongest corollaries of input milk matched with a High-Bif_qPCR_ stool were high absolute amounts of non-fucosylated neutral HMOs, high absolute amounts of α(1-2)-fucosylated HMOs, and low relative amounts of α(1-3/4)-fucosylated HMOs, perhaps reflecting competitiveness with 2′-fucosylation. This may be due to adaptations of infant-type bifidobacteria to efficiently and selectively consume fucosylated and undecorated oligosaccharides [11,12,52,53]. Our data also provide insight into the metabolism of input milk glycans by different types of microbiota. BI_PCoA_ feces possessed fewer oligosaccharides of all types, suggesting that these populations are capable of metabolizing greater amounts of fucosylated, sialylated, and undecorated sugars than communities low in bifidobacteria.

Some environmental pressures (pH, carbon source availability, and so on) that select for bifidobacteria likely select for other taxa as well. It is important to note that relative abundances (such as provided by most marker gene sequencing workflows) are a zero-sum game; when one taxa’s relative abundance increases, it registers as a concurrent decrease in other taxa. For some communities, this type of data may not adequately describe the underlying ecological interactions. This is illustrated in our study by the correlation difference between the relative and absolute bifidobacterial abundances with *Bacteroidales* in secretor-fed infants (−0.3 with relative abundance; 0.2 with absolute abundance). The relative abundance data seem to indicate that *Bacteroides* is antagonistic to (negatively correlated with) bifidobacteria, while the absolute abundance (qPCR) data show that they are mildly positively correlated with each other. It may be that bifidobacteria and *Bacteroides* populations respond to some environmental conditions in the same way but not to the same magnitude. The presence of HMGs that both genera can consume may be an example of this [54]. Both genera were enriched in secretor-fed infants, but the amplitude of the response as shown by the correlation matrix, however, appears to differ. This fact would be disguised by relative abundance data alone.

Bifidobacteria and *Bacteroides* were implicated here as the major HMO consumers in infants, which agrees with previous *in vitro* work [55]. *Bacteroides*-dominated communities had a lower percentage of undecorated HMOs remaining, suggesting that they consume undecorated sugars preferentially to at least some decorated sugars. Our data show that fucosylated sugars remain at a significantly higher level (relative to other sugars) by *Bacteroides*-dominated feces, implying that they are not a preferred substrate of members of that taxa. Bifidobacteria-dominated feces have lower absolute amounts of fucosylated oligosaccharides than *Bacteroides*- and OT-dominated communities. This suggests that fucosylated oligosaccharides might enrich bifidobacteria more than *Bacteroides*, supporting the difference shown in the correlation matrix between absolute and relative abundance of bifidobacteria and *Bacteroides* in secretor-fed infants. However, it may be that only some bifidobacterial species would possess this advantage, due to differences in abilities of various species to consume fucosylated substrates.

In aggregate, the bifidobacterial species distribution did not differ greatly between secretor-fed infants and non-secretor-fed infants. As the species of bifidobacteria present within an infant are dependent on environmental exposure, we tentatively conclude that this subset of geographically co-located infants was exposed to similar sets of bifidobacterial species. However, there was a large difference in which species of bifidobacteria thrived in each group of infants (defined as a species whose presence tended to lead to domination of the microbiota by bifidobacteria). A broader range of bifidobacterial species were positively correlated with the amounts of overall bifidobacteria within secretor-fed infants than non-secretor-fed infants, suggesting that the presence of 2′-fucosylated sugars allows a broader array of bifidobacterial species to colonize the gut environment. According to our fecal isolate data, bifidobacteria that can grow on 2′-fucosylated substrates are relatively uncommon, but represent a larger proportion of total bifidobacteria in secretor-fed infants, suggesting that this substrate is related to the competitive fitness of these strains. Previous work supports the idea that fucosidases are a differentiating factor in the ability to grow on 2′-fucosylated sugars, although further study is needed to definitively identify which class(es) of fucosidase(s) is/are necessary [10].

That *B. longum* subsp. *infantis* thrives in secretor-fed infants is no surprise. It possesses both classes of fucosidases (GH95 and GH29) and was shown here to grow on 2′-fucosyllactose *in vitro* [10]. Feces dominated by this subspecies also had lower percentages of fucosylated oligosaccharides remaining. However, why *B. longum* subsp. *infantis* failed to dominate in any non-secretor-fed infant is somewhat perplexing. It may be that *B. longum* subsp. *infantis* specializes in consuming 2′-fucosylated oligosaccharides to gain an advantage over other species. *B. breve* on the other hand seems to be an oligosaccharide generalist, as it was dominant in examples of infants fed by both types of milk. *B. breve* strains are known to be variable in their capacity to consume 2′-fucosylated oligosaccharides [36].

The fact that *B. breve* and not *B. longum* subsp. *infantis* seems to thrive in non-secretors may account for the observation of Avershina *et al.* that *B. breve* abundance separated the 10-day-old infants in their study into two groups; one group where it accounted for <15%, whereas in the other it accounted for >75% of the bifidobacterial load. *B. longum* group members were dominant among all infants in that study [56]. The *B. breve*-dominated group may correspond to a non-secretor-fed infant minority in their study. As they did not track the secretor status of the mothers in their study, this remains an open question. It is also important to note that their cohort was located in Norway and that the bacterial exposure pattern may differ from that of our cohort. The differing levels of competitiveness of *B. breve* and *B longum* subsp. *infantis* in infants fed by mothers of differing secretor status may also account for the observation that a three-probiotic mixture containing both *B. breve* and *B longum* subsp. *infantis* was more effective at promoting high levels of bifidobacteria in breastfed premature infants than a probiotic containing *B. breve* alone [57].

The bimodal distribution of bifidobacterial abundance shown by qPCR is intriguing from an ecological perspective. A remarkably similar bimodal distribution with comparable ranges was found by Mikami *et al.*, suggesting that this phenomena may be widespread [58]. According to this distribution, a useful cutoff value for defining the population level of bifidobacteria in an infant as high or low would be around 10^8^/g of feces. Using this cutoff, our data also agree with the growing consensus that the establishment of bifidobacteria can happen in many infants in the first week of life [59,60].

The ecological phenomena of alternative stable community states may help explain this bimodal distribution in the infant gut. Simply stated, alternative stable state theory posits that a change in an ecosystem or in environmental conditions can result in a drastic shift in the composition of a community once some threshold or breakpoint is reached (review of concept here [61]). Some bifidobacteria are known to produce bacteriocins, which could contribute environmental pressure to maintaining alternate stable community states, once a threshold number of bifidobacteria producing these bacteriocins is reached [62]. A more likely mechanism by which these alternative stable states might be formed in this environment involves the production of lactate and short-chain fatty acids (SCFA) and their influence on environmental pH. Perhaps once a certain threshold of bifidobacteria/gram feces is reached the amount of SCFA and lactate produced alters the pH of the gut lumen, overcoming the buffering capacity of the luminal contents. Due to the lower pH, a shift in the community structure could occur as non-acid-tolerant members of the community die off. It is known that pH is a major driver in the composition of soil microbial communities [63-66]. The same physiological constraints that select for microorganisms able to grow at a low pH in soil likely apply in gut communities as well. One of these constraints may be effect of environmental pH on intracellular pH homeostasis and proton motive force [67]. *Bacteroides* in particular is known to be sensitive to low-pH conditions, and the sensitivity is increased in the presence of SCFAs [68-71]. At least some enterobacteria are known to be pH sensitive as well, which may indicate that a low-pH gut is protective against infectious disease [70]. A decrease in the abundance of other microbes due to pH changes, especially a decrease in competitors for HMG substrates, such as *Bacteroides* [54,72], would allow bifidobacteria to thrive from the reduced competition for nutrients and space.

A survey of breastfed and formula-fed infants showed that fecal pH and SCFAs were lower in breastfed infants (pH mean 5.8) than formula-fed infants (pH mean 7.1), but that lactic acid was higher, suggesting that lactic acid might be a driver of pH [73]. Lactic acid has a lower pKa than the common SCFAs and would be hypothesized to have a greater effect on pH. *B. longum* subsp. *infantis* grown on HMOs was found to produce higher molar amounts of acetate and lactate [74]. Breastfed infants are known to have higher levels of bifidobacteria than formula-fed infants and also have different types of bifidobacteria, which may be important to the SCFA profile [75,76]. Both *Bacteroides* and bifidobacteria are known to produce lactate, which may explain the difference we observed between the amount of lactate in feces dominated by those groups as opposed to other species [77]. Although our data showed that both bifidobacteria- and *Bacteroides*-dominated fecal communities were higher in lactic acid than communities dominated by other species, our methods did not test the flux of lactate production and utilization by both the microbiota and the host. Nevertheless, lactate levels may be an interesting biomarker of the composition of the microbial community of the infant gut.

There are numerous ways in which the composition of the gut microbiota impacts health. In the first 2 years of life, the infant gut microbiome progresses through a series of age-associated taxonomic changes. Infants and their gut microbiota are sensitive to disruptions during the early days and weeks of life [1,78,79]. Indeed, recent evidence suggests infants suppress the immune system early in life to aid in developing a healthy microbiota [80]. Like any new ecosystem, the microbial community in the gut has unfilled niches and has not developed mature levels of colonization resistance [81]. The establishment of the gut microbiota can impact an individual’s lifelong health, and an early intervention that makes beneficial changes could have lifelong positive effects [82,83].

Early establishment of bifidobacteria is thought to be beneficial in numerous ways. Recently, domination by bifidobacteria was shown to be associated with improved immune response to vaccines [5]. Other benefits include protection from pathogens and development of the neonatal immune system [3,5,84,85]. For these reasons, our finding of delayed bifidobacterial colonization of non-secretor-fed infants (despite a higher incidence of exclusive breastfeeding in non-secretors) has important implications. Understanding the mechanism behind these differences will prove crucial to potentially compensating for this problem, perhaps through carefully chosen prebiotics and/or probiotics.

It may be that the deficit in bifidobacteria that non-secretor-fed infants experience is due to the likelihood of acquiring a species of bifidobacteria with metabolic abilities appropriate for the type of milk being consumed. As colonization by bifidobacteria is thought to be dependent on stochastic exposure to environmental strains, fewer appropriate potentially colonizing species may mean a lower likelihood of obtaining an appropriate one, whatever the mechanism of acquisition. As modern hygiene standards and other cultural practices may lead to a reduction in the exposure levels to different bifidobacterial species, the phenomenon of the low bifidobacteria infant might be an artefact of developed countries. Indeed, recent studies of developing areas of the world have revealed widespread domination of bifidobacteria in infants [5,86,87]. More surveys of the absolute abundance of infant bifidobacteria in developing and undeveloped areas of the world are needed, along with the measurement of relevant exposure-related metadata.

## Conclusion

In conclusion, our work reveals important functional differences in the microbiota of infants fed by mothers of differing secretor. This knowledge will be useful to those selecting bifidobacterial species for probiotic interventions in breastfed infants [88]. As a mother’s secretor status can be determined relatively easily, it could be used as a marker to target clinical interventions administering probiotics to infants to match the set of glycans the mother provides. This work provides context and insights for future hypothesis testing related to the *in vivo* competition between bifidobacteria and other members of the microbiota, as well as among bifidobacterial species. Further work is necessary to determine if these apparent differences in bifidobacterial populations between secretor phenotypes are indeed a developed-world phenomenon, and if the ‘hygiene hypothesis’ mechanism we propose here plays a role.

## Methods

### Subjects

Milk samples were obtained from 44 healthy women enrolled in the Foods for Health Institute Lactation Study at UC Davis. Subjects were enrolled at approximately 34 weeks of gestation and asked to fill out detailed health history questionnaires regarding demographics, anthropometrics, pregnancy history, current and prior health history, dietary habits and restrictions, physical activity level, as well as medication and supplementation intake history. Subjects reported the mode of delivery of their infants (C-section *versus* vaginal), infant sex, weight, length, and gestational age at birth, and filled out questionnaires regarding the health of themselves and their infants, as well as their diet throughout the study. Subjects received lactation support and training on proper sample collection from the study’s lactation consultant. The UC Davis Institutional Review Board approved all aspects of the study, and informed consent was obtained from all subjects. This trial was registered on clinicaltrials.gov (ClinicalTrials.gov Identifier: NCT01817127).

### Breast milk samples

Subjects were instructed to write on all sample tubes the time, date of collection, time of last meal prior to collection, and contents of the meals. Milk samples were collected in the morning on day 6, 21, 71, and/or 120 postpartum using a modified published method [89] involving milk collection by the subject from one breast using a Harmony Manual Breastpump (Medela Inc., McHenry, IL, USA) 2 to 4 h after feeding her infant. Subjects fully pumped one breast into a bottle, inverted six times, transferred 12 ml into a 15-ml polypropylene tube, and subsequently froze the sample in their kitchen freezers (−20°C). Samples were picked up biweekly, transported to the lab on dry ice, and stored at −80°C until processing.

### Infant stool samples

Infant fecal samples were collected from the 44 breastfed-term infants born to women in the study at 6, 21, 71, and/or 120 days of life. Only one of the infants enrolled in this study had received antibiotic treatment at 89 days postpartum. All of the infants consumed breast milk, and several infants also consumed infant formula or solid food throughout the study duration. Parents were prompted to fill out detailed labels on each stool sample vial regarding infant intake of solids, infant formula, medications, and supplements. Parents transferred their infant fecal samples into sterile plastic tubes and were instructed to immediately store the samples in −20°C until transported by study personnel. Fecal samples were transported to the laboratory on ice packs and stored at −80°C before processing.

### Infant metadata statistics

Differences in demographics and characteristics between secretor and non-secretor women were analyzed by the Pearson chi-squared test. Differences in clinical characteristics between secretor and non-secretor women were analyzed non-parametrically for unequal sample sizes using Mann-Whitney *U* test. Alpha was set at 0.05.

### Oligosaccharide extraction from milk and stool

Glycans were extracted from 50 μl of breast milk that was aliquoted onto two 96-well plates. Milk was defatted *via* centrifugation; the skimmed milk was collected and subjected to an ethanol precipitation for the removal of proteins. Following protein precipitation, the liquid fraction containing the oligosaccharides was dried completely using centrifugal evaporation. The oligosaccharides were reconstituted in 50 μl of water and reduced to their alditol forms with 1 M NaBH_4_. This is done in order to eliminate alpha and beta anomers on the reducing end of the sugars. Following reduction, the oligosaccharide mixture was desalted and enriched by solid-phase extraction on graphitized carbon-packed 96-well plates. Samples were desalted with six column volumes (approximately 1.2 ml) of deionized water and eluted with 20% acetonitrile in water, followed by 40% acetonitrile and 0.01% trifluoroacetic acid in water. Eluent was dried completely, reconstituted with 50 μl of deionized water, and diluted 50 fold for liquid chromatography-mass spectrometry (LC-MS) analysis.

Glycans were extracted from 50 mg of homogenized stool. Stool was diluted to 100 mg/ml with deionized water. Diluted stool samples were then homogenized by rocking the vials overnight. The solid components of the stool were then separated by centrifugation, then 100 μl of the oligosaccharide-rich supernatant was aliquoted onto two 96-well plates. Two times the sample volume (approximately 300 μl) of ethanol was added to each well. Proteins were precipitated at −80°C for 1.5 h, and then centrifuged at 3,220 rcf for 30 min at 4°C. The supernatant containing the HMOs was then collected and dried completely. Samples were reconstituted in 100 μl of deionized water and reduced with 100 μl of 2 M NaBH_4_ (1 M final concentration in 200 μl solution total). Reduction was performed in a 65°C water bath for 1.5 h. Samples were immediately transferred to C8 packed 96-well plates for removal of residual proteins and peptides by solid-phase extraction. The C8 flow-through containing the oligosaccharides was then desalted and enriched by solid-phase extraction on graphitized carbon 96-well plates. Samples were desalted and enriched following the same protocol for milk as described above. Eluted glycans were then dried down, reconstituted in 100 μl of deionized water, and diluted tenfold for LC-MS analysis.

### Glycoprofiling by nano-LC chip TOF (time of flight) mass spectrometry

Both milk and fecal HMOs were analyzed using an Agilent nano-LC Chip time-of-flight mass spectrometer (Agilent, Santa Clara, CA, USA), as described previously [32,90]. Briefly, all chromatography was done on a nano-scale microfluidic chip, equipped with a trapping column for sample enrichment and an analytical column for separation, both packed with porous graphitized carbon (PGC). Directly from the 96-well plate, 1 μl of HMO sample was injected and loaded onto the enrichment column and subsequently separated on the analytical column with a gradient optimized for glycan separation, using 3% acetonitrile and 0.1% formic acid in water as aqueous solvent A and 90% acetonitrile and 0.1% formic acid in water as organic solvent B (gradient also described previously). LC-MS data was deconvoluted using Agilent’s MassHunter Qualitative Analysis software, version B.03.01 (Agilent, Santa Clara, CA, USA). Oligosaccharides were identified by matching retention time and exact mass to a fully annotated, in-house HMO library [90,91].

### Oligosaccharide quantitation and statistics

Total oligosaccharide abundance was determined for each sample by summing the signal of all identified HMO peaks (in ion counts). Oligosaccharides were then grouped by glycan class, designated as either fucosylated, sialylated, or non-fucosylated neutral (containing neither fucose nor sialic acid residues). Total absolute amounts of fucosylation and sialylation were determined by summing the abundance (peak volume in ion counts) of all HMOs containing either fucose or sialic acid residues, respectively. Relative amounts for each glycan class were determined by normalizing absolute abundance of each class to the total HMO signal and were expressed as percentages. Two-tailed, unpaired *t*-test, with an α of 0.05, was used to compare glycan expression in milk between secretor and non-secretor mothers, as well as comparing milk and fecal HMOs received by infants with low amounts of bifidobacteria *versus* those with high amounts of bifidobacteria, as defined in the *Bifidobacterium*-specific qPCR section below. ANOVA was used to compare fecal glycan expression between infants whose dominant bacterial species were categorized as *Bacteroides*, bifidobacteria, or ‘other’, by PCoA grouping (explained below) followed by a pairwise comparison of means between each of the aforementioned groups using Tukey-Kramer HSD test, with an α of 0.05.

### Bifidobacterial isolations

To isolate bifidobacteria, 100 mg of each fecal sample was aseptically transferred to a sterile tube, diluted tenfold with sterile phosphate buffered saline (PBS), and homogenized by vortex. Serial dilutions were prepared in PBS and inoculated on modified *Bifidobacterium* selective iodoacetate mupirocin (BSIM) agar. Modified BSIM agar was prepared by supplementing de Man Rogosa Sharpe (MRS) media with 13 g/l agar, 500 mg/l of l-cysteine-HCL, 20 mg/l of nalidixic acid, 50 mg/ml mupirocin, 50 mg/ml kanamycin, 50 mg/ml polymixin B sulfate, 100 mg/ml Iodoacetate, and 100 mg/ml 2,3,5-triphenyltetrazolium chloride. The plates were inoculated for 48 h at 37°C in an anaerobic chamber with an atmosphere containing approximately 5% carbon dioxide, 3% hydrogen, and the remainder nitrogen. Up to ten resulting colonies from each sample with the appropriate colony appearance were streaked onto BSIM plates for purity for two passages. The resulting pure strains were grown in MRS broth supplemented with 0.05% l-cysteine and stored at −80°C in 50% glycerol.

### MALDI-TOF Biotyper MS identification of isolates

Glycerol stocks of each isolate were streaked on MRS plates and incubated at 37°C for 48 h in an anaerobic chamber. A colony from each plate was added to 300 μl nuclease-free water in a microcentrifuge tube and homogenized by vortex. Next, 900 μl of 98% ethanol was added to the tube, pulse vortexed, and centrifuged for 2 min at maximum speed. The supernatant was removed and the tubes were centrifuged again for 2 min. All liquid was removed from the pellets, and the samples were left at room temperature to allow the ethanol to evaporate. Subsequently, 25 μl of formic acid was then added to each tube and homogenized by vortex, followed by the addition of 25 μl of acetonitrile. Samples were then centrifuged for 2 min, and 1 μl of extract was placed on a MALDI target plate, left to dry at room temperature, covered with an α-Cyano-4-hydroxycinnamic acid (HCCA) matrix, and air dried. The MALDI target plate was then analyzed by a MALDI Biotyper (Bruker, Fremont, CA, USA), and the best database match for each isolate was recorded.

### Bacterial *in vitro* growth on 2′-fucosyllactose

Unique bifidobacterial isolates (one isolate of each identified species from each infant fecal sample) were tested for growth on modified MRS (mMRS) with 3% filter-sterilized 2′-fucosyllactose as the sole carbon source, using mMRS with 3% lactose as a sole carbon source as a positive control and a no-sugar mMRS as a negative control. mMRS contains 10 g/l bacto-peptone, 5 g/l yeast extract, 2 g/l dipotassium phosphate, 5 g/l sodium acetate, 2 g/l ammonium citrate, 200 mg/l magnesium sulfate, 50 mg/l manganese sulfate, 5 g/l beef extract, 500 mg/l cysteine-HCL, and 1,000 g/l Tween 80. *B. infantis* ATCC 15697 and *B. animalis* UCD316 were included as positive and negative growth controls, respectively. Isolates were streaked from glycerol stock onto reinforced clostridial media (RCM) plates and incubated at 37°C in an anaerobic chamber for 48 h. A resulting colony was inoculated in 1 ml RCM broth at 37°C in an anaerobic chamber for 16 h. Five microliters of each resulting overnight culture was used to inoculate 100 μl of mMRS medium supplemented with either 3% (*w*/*v*) of 2′-fucosyllactose, 3% (*w*/*v*) of lactose, or mMRS without added sugar. The cultures were grown in 96-well microtiter plates in triplicate and covered with 20 μl of sterile mineral oil to avoid evaporation. The incubations were carried out at 37°C in an anaerobic chamber. Cell growth was monitored by OD at 600 nm every 30 min preceded by 30 s of shaking at a variable speed for a total of 96 h. The OD obtained for each of the technical triplicates from each strain grown on each substrate was averaged together and compared to the OD obtained in the absence of sugar source. This difference in OD (ΔOD) was used as a parameter to evaluate the strain’s ability to grow on the different substrates. 2′-fucosyllactose was produced as described previously [92].

### Fecal DNA extraction

DNA was extracted from 150 mg of stool sample using the ZR Fecal DNA MiniPrep kit (ZYMO, Irvine, CA, USA) in accordance with the manufacturer’s instructions, which included a bead-beating step using a FastPrep-24 Instrument (MP Biomedicals, Santa Ana, CA, USA) for 2 min at 25°C at a speed of 6.5 m/s.

### Breast milk and saliva DNA extraction

DNA was extracted from breast milk for the secretor genotyping assay using the Qiagen DNeasy Blood and Tissue kit (Qiagen, Venlo, Netherlands) with a modified protocol for extracting DNA from animal saliva obtained from the Qiagen website. Briefly, 2 ml of breast milk or saliva was spun in a microcentrifuge at 15,000 rpm for 30 min to pellet human cells. Cells were washed once in PBS and re-pelleted. The pellet was re-suspended in 180 μl of PBS and incubated with 25 μl of proteinase K and 200 μl of buffer AL for 10 min at 56°C. Two hundred microliters of ethanol was added to the sample and mixed by vortexing. The entire sample was loaded onto a spin column, and purification proceeded as per the manufacturer’s recommended protocol from that point. DNA was eluted in 30 μl of buffer EB for increased concentration.

### Determination of secretor genotype

Genomic DNA purified from each mother’s breast milk or saliva was amplified with primers FUT2-F (5′-CCTGGCAGAACTACCACCTG) and FUT2-R (5′-GGCTGCCTCTGGCTTAAAGA), which produces a 608-bp amplicon. Each reaction contained 25 μl of 2X GoTaq Green master mix (Promega, Madison, WI, USA), 5 μl of DNA, 1 μl of each primer (10 μM), 6 μl of MgCl_2_ (25 mM), and 12 μl of nuclease-free water. Cycling conditions were 95°C for 2 min followed by 35 cycles of 95°C for 1 min, 60°C for 1 min, and 72°C for 1 min. A final elongation was allowed at 73°C for 5 min, after which the products were kept at 4°C overnight. Successful amplification was confirmed by gel electrophoresis, and the PCR products were purified using the QIAquick PCR purification kit (Qiagen) according to the manufacturer’s instructions. Samples with low amplicon concentrations were attempted again with 50 cycles of PCR, which was successful in amplifying difficult samples. The amplicons were then digested with BfaI, which cuts the DNA of individuals containing the mutated non-secretor rs601338 SNP (G → A, Trp → Ter) allele of the FUT2 gene, the predominant non-secretor mutation in the U.S. The resulting digests were electrophoresed on a 2% agarose gel for approximately 2 h at 80 V, and the resulting bands were visualized using GelGreen dye (Biotum, Hayward, CA, USA) under UV light. Individuals possessing a secretor allele produce a 608-bp band on the gel after digestion, while a non-secretor allele produces two bands with sizes of 516 and 92 bp. In this way, it is possible to easily distinguish both homozygote genotypes from each other and from heterozygotes.

### Determination of secretor phenotype

The mother’s secretor status phenotype in milk was determined by quantitating fucosylated glycan markers that have been previously described and assessed for sensitivity and specificity [32]. Secretor status was determined once per mother using milk from the earliest available time point, as the influence of early milk is thought to be most influential in establishing microbiota [6]. Thus, by our definition, ‘secretor’ and ‘non-secretor’ might be thought of as ‘early secretor’ or ‘early non-secretor’ since the phenotypes were defined at an early time point. Among these markers are α(1-2)-fucosylated structures, including 2′-fucosyllactose (2′FL, m/z 491.19), lactodifucotetraose (LDFT, m/z 637.25), lacto-N-fucopentaose I (LNFP I, mz/ 856.33), isomeric fucosylated lacto-N-hexaose (IFLNH I, m/z 1221.45), difucosyllacto-N-hexaose a (DFLNH a, m/z 1367.51), and difucosyllacto-N-hexaose c (DFLNH c, m/z 1367.51). Cutoff values for the relative amounts of each marker were used to distinguish secretor women from non-secretor women, as described previously [32].

### Bif-TRFLP

The method of Lewis *et al.* was used to perform the *Bifidobacterium*-specific terminal restriction fragment length polymorphism assay [93]. Briefly, DNA from feces was amplified in triplicate by PCR using primers NBIF389 (5′-[HEX]-GCCTTCGGGTTGTAAAC) and NBIF1018 REV (GACCATGCACCACCTGTG). DNA was purified using the Qiagen QIAquick PCR purification kit and then cut with restriction enzymes AluI and HaeIII. The resulting fragments were analyzed on an ABI 3100 genetic analyzer, and sizes were compared against the published database for species identification.

### *Bifidobacterium longum*/*infantis* ratio (BLIR)

A PCR-based assay, BLIR, was developed in order to determine which subspecies of *B. longum* were present in each sample and to gain an estimate of their relative abundance to each other. Three primers (FWD_BL_BI (5-[HEX]-AAAACGTCCATCCATCACA), REV_BL (5-ACGACCAGGTTCCACTTGAT), and REV_BI (5-CGCCTCAGTTCTTTAATGT)) were designed to target a conserved portion of the genome (between Blon_0424 and Blon_0425) shared by both subspecies using multiple genome sequences of each subspecies. FWD_BL_BI is complementary to a sequence in both subspecies, while REV_BL and REV_BI are complementary to nearby sequences in only *B. longum* subsp. *longum* and *B. longum* subsp. *infantis*, respectively. FWD_BL_BI and REV_BL amplify a fragment of the *B. longum* spp. *longum* genome 145 bp in length, while FWD_BL_BI and REV_BI amplify a fragment of the *B. longum* subsp. *infantis* genome 114 bp in length.

DNA from each sample was amplified by PCR using 0.5 μl of 10 μM stock of each of the above primers, 12.5 μl GoTaq Green Master Mix (Promega), 1 μl of 25 mM MgCl_2,_ 1 μl of template DNA, and 9 μl of nuclease free water. Cycling conditions were 95°C for 2 min, 30 cycles of 95°C for 1 min, 54°C for 1 min, and 72°C for 30 seconds, followed by a 72°C extension for 5 min. PCR products were purified from the mixture using the QIAquick PCR purification kit (Qiagen), and diluted 1:10. 1.5 μl of the dilutions were analyzed by capillary electrophoresis on an ABI 3100 genetic analyzer (Applied Biosystems, Carlsbad, CA, USA). The HEX fluorophore (Abcam, Cambridge, UK) on the common primer allowed detection and differentiation of amplicon sizes and a rough quantitation of the abundance of each amplicon based on peak area when the samples were analyzed with PeakScanner 2.0 software (Applied Biosystems, Carlsbad, CA). A positive control was included with each PCR run to ensure potential amplification of both *B. longum* subsp. *longum* and *B. longum* subsp. *infantis* products.

### *Bifidobacterium* qPCR

Levels of *Bifidobacterium* were measured by qPCR using the methods of Penders *et al.* and performed on a 7500 Fast Real-Time PCR System (Applied Biosystems) [94]. All reactions were carried out in triplicate with a non-template control and compared to a standard curve with known quantities of bifidobacterial DNA.

### Marker gene sequencing

DNA samples were prepared for 16S rRNA marker gene sequencing as previously described [95] with the following modifications. Universal barcoded primers with Illumina sequencing adapters (Illumina, San Diego, CA, USA) (adapters are italicized and the barcode is highlighted in bold) V4F (5′-*AATGATACGGCGACCACCGAGATCTACACTCTTTCCCTACACGACGCTCTTCCGATCT***NNNNNNNN**GTGTGCCAGCMGCCGCGGTAA-3′) and V4Rev (5′-*CAAGCAGAAGACGGCATACGAGATCGGTCTCGGCATTCCTGCTGAACCGCTCTTCCGATCT*CCGGACTACHVGGGTWTCTAAT-3′) were used to PCR amplify the V4 region of the 16S rRNA gene [95]. PCR reactions were carried out in triplicate and contained 12.5 μl 2X GoTaq Green Master Mix (Promega, Madison, WI, USA), 1.0 μl 25 mM MgCl_2_, 8.5 μl water, 0.5 μl forward and reverse primers (10 μM final concentration), and 2.0 μl DNA. The triplicate reactions were combined and cleaned, and DNA concentrations were quantified using the PicoGreen dsDNA kit (Thermo Fisher Scientific, Waltham, MA, USA). An equimolar composite sample mixture was made, gel purified, and sequenced at the University of California DNA Technologies Core Facility on an Illumina MiSeq sequencing platform (Illumina) (150 bp single read).

### Sequence analysis

The QIIME software package (version 1.7.0) was used to analyze the results of the Illumina sequencing run. Illumina V4 16S rRNA gene sequences (Illumina) were demultiplexed and quality filtered using the QIIME 1.7.0 software package with default settings unless otherwise specified [96]. Reads were truncated after a maximum number of three consecutive low quality scores. The minimum number of consecutive high-quality base calls to include a read (per single end read) as a fraction of the input read length was 0.75. The minimum acceptable Phred quality score was set at 20. Similar sequences were clustered into operational taxonomic units (OTUs) using open reference OTU picking with UCLUST software [97]. Taxonomy was assigned to each OTU with the Ribosomal Database Project (RDP) classifier [98] and the RDP taxonomic nomenclature [99]. OTU representatives were aligned against the Greengenes core set [100] with PyNAST software [101]. PCoA plots were generated using the default beta diversity analysis parameters.

### Lactate concentrations

Lactate concentration was measured in a modified version of the procedure developed by Ford *et al.* [102]. Shortly, lactate was extracted from solid feces by agitation in a 12-fold excess of pH 5.5 phosphate buffer for 3 h at 4°C. Proteins were removed by ethanol precipitation, and 100 μl of each extract was collected for analysis. After spiking the samples with stable isotope standards, 0.2 M aqueous 3-Ethyl-1-[3-(dimethylamino)propyl]carbodiimide was used to link an excess of 2-phenyl-2-ethanamine to the fatty acids *via* a peptide bond. The reaction was run for 20 min at room temperature and quenched by an ice bath followed by immediate C18 solid-phase extraction. The extracted phenylethylamine adducts were dried in a vacuum and reconstituted in water.

The aqueous derivatives were analyzed on an Agilent 6490 QQQ LC/MS system (Agilent), and the response was gauged by characteristic transitions due to the Y and B fragmentation of the peptide bonds. Quantitation was achieved by comparison of the derivatized analytes to internal stable isotope standards. Lactate was quantified by comparison to 3,3,3-D_3_-lactate. During statistical analysis, the Bartlett test was used to test for homoscedasticity, and when needed, data was log transformed to meet the assumptions required to conduct a parametric test. For pairwise comparisons, two-tailed Student’s *t*-tests were used. Results were determined to be significant for *p* < 0.05.

## Additional files

Additional file 1: Table S1. Markers used for the determination of secretor status. Raw ion counts and normalized sum of oligosaccharide markers used to define secretor phenotype. Averages for each group are included. Additional file 2: Table S2. Breast milk and infant fecal sampling from mother-infant dyads per time point. ^1^Self report by parent who was prompted to answer the question ‘has your infant consumed any solids or infant formula’ with each stool sample. The days of life listed include each day when solids or infant formula intake was reported. Additional file 3: Figure S1. C-section (*N* = 5) *versus* vaginal (*N* = 39) birth over time. Shows composition of the microbiota as determined by marker gene sequencing. Additional file 4: Figure S2. Example growth curves of bifidobacterial growth on 2′-fucosyllactose. One representative growth curve for each level of OD (high ≥ 0.8, medium = 0.4 to 0.8, low ≤ 0.4) is shown here. Data in Figure 13 shows the breakdown of all unique isolates by this assignment method. Additional file 5: Table S3. BIO-ENV analysis. Taxa used for evaluation of importance to overall variation between microbial communities of samples, along with iterative results adding in the next most important group and respective Rho statistics for each iteration. Additional file 6: Figure S3. PCoA plots of the NGS data. Colored by days after birth (left), assigned group (center) and mother’s secretor phenotype (right). Additional file 7: Figure S4. PCoA plots of the NGS data. Colored by the abundance of *Bifidobacterium* (left), Bifidobacteria from qPCR data (center) and *Bacteroides* (right). Colors represent a spectrum of abundance, with blue being high and red being low. Additional file 8: Figure S5. PCoA plots of the NGS data. Colored by the abundance of ‘Clostridiaceae_other’ (top left), *Escherichia*/*Shigella* (top right), *Veillonella* (bottom left), and *Streptococcus* (bottom right). Colors represent a spectrum of abundance, with blue being high and red being low. Additional file 9: Figure S6. PCoA plots of the NGS data. Colored by the abundance of ‘Enterobacteriaceae_other’ (top left), *Streptococcus* (top right), *Escherichia*/*Shigella* (bottom left), and ‘Clostridiaceae_other’ (bottom right). Colors represent a spectrum of abundance, with blue being high and red being low. Additional file 10: Table S4. ANOSIM analysis. ANOSIM R statistics and *p* values for various ways of grouping samples.
