# Supplementary material for: Maternal fucosyltransferase 2 status affects the gut bifidobacterial communities of breastfed infants
Source: Microbiome. 2015 Apr 10;3:13. doi: 10.1186/s40168-015-0071-z (PMC4412032; doi:10.1186/s40168-015-0071-z)
Supplement: Additional file 1: Table S1. — Markers used for the determination of secretor status. Raw ion counts and normalized sum of oligosaccharide markers used to define secretor phenotype. Averages for each group are included. [file 40168_2015_71_MOESM1_ESM.pptx]

## Slide 1
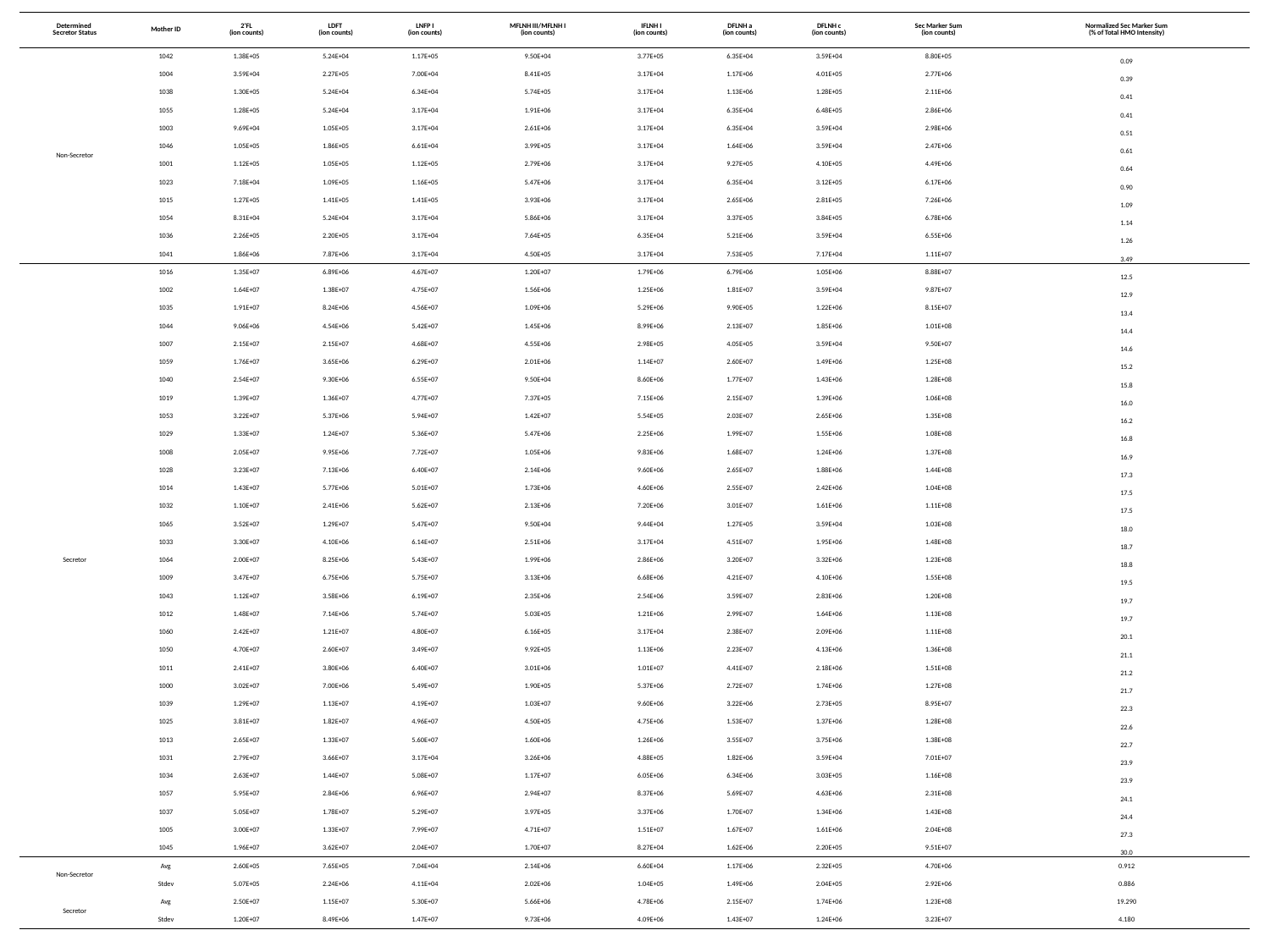

| Determined Secretor Status | Mother ID | 2'FL (ion counts) | LDFT (ion counts) | LNFP I (ion counts) | MFLNH III/MFLNH I (ion counts) | IFLNH I (ion counts) | DFLNH a (ion counts) | DFLNH c (ion counts) | Sec Marker Sum (ion counts) | Normalized Sec Marker Sum (% of Total HMO Intensity) |
| --- | --- | --- | --- | --- | --- | --- | --- | --- | --- | --- |
| Non-Secretor | 1042 | 1.38E+05 | 5.24E+04 | 1.17E+05 | 9.50E+04 | 3.77E+05 | 6.35E+04 | 3.59E+04 | 8.80E+05 | 0.09 |
| | 1004 | 3.59E+04 | 2.27E+05 | 7.00E+04 | 8.41E+05 | 3.17E+04 | 1.17E+06 | 4.01E+05 | 2.77E+06 | 0.39 |
| | 1038 | 1.30E+05 | 5.24E+04 | 6.34E+04 | 5.74E+05 | 3.17E+04 | 1.13E+06 | 1.28E+05 | 2.11E+06 | 0.41 |
| | 1055 | 1.28E+05 | 5.24E+04 | 3.17E+04 | 1.91E+06 | 3.17E+04 | 6.35E+04 | 6.48E+05 | 2.86E+06 | 0.41 |
| | 1003 | 9.69E+04 | 1.05E+05 | 3.17E+04 | 2.61E+06 | 3.17E+04 | 6.35E+04 | 3.59E+04 | 2.98E+06 | 0.51 |
| | 1046 | 1.05E+05 | 1.86E+05 | 6.61E+04 | 3.99E+05 | 3.17E+04 | 1.64E+06 | 3.59E+04 | 2.47E+06 | 0.61 |
| | 1001 | 1.12E+05 | 1.05E+05 | 1.12E+05 | 2.79E+06 | 3.17E+04 | 9.27E+05 | 4.10E+05 | 4.49E+06 | 0.64 |
| | 1023 | 7.18E+04 | 1.09E+05 | 1.16E+05 | 5.47E+06 | 3.17E+04 | 6.35E+04 | 3.12E+05 | 6.17E+06 | 0.90 |
| | 1015 | 1.27E+05 | 1.41E+05 | 1.41E+05 | 3.93E+06 | 3.17E+04 | 2.65E+06 | 2.81E+05 | 7.26E+06 | 1.09 |
| | 1054 | 8.31E+04 | 5.24E+04 | 3.17E+04 | 5.86E+06 | 3.17E+04 | 3.37E+05 | 3.84E+05 | 6.78E+06 | 1.14 |
| | 1036 | 2.26E+05 | 2.20E+05 | 3.17E+04 | 7.64E+05 | 6.35E+04 | 5.21E+06 | 3.59E+04 | 6.55E+06 | 1.26 |
| | 1041 | 1.86E+06 | 7.87E+06 | 3.17E+04 | 4.50E+05 | 3.17E+04 | 7.53E+05 | 7.17E+04 | 1.11E+07 | 3.49 |
| Secretor | 1016 | 1.35E+07 | 6.89E+06 | 4.67E+07 | 1.20E+07 | 1.79E+06 | 6.79E+06 | 1.05E+06 | 8.88E+07 | 12.5 |
| | 1002 | 1.64E+07 | 1.38E+07 | 4.75E+07 | 1.56E+06 | 1.25E+06 | 1.81E+07 | 3.59E+04 | 9.87E+07 | 12.9 |
| | 1035 | 1.91E+07 | 8.24E+06 | 4.56E+07 | 1.09E+06 | 5.29E+06 | 9.90E+05 | 1.22E+06 | 8.15E+07 | 13.4 |
| | 1044 | 9.06E+06 | 4.54E+06 | 5.42E+07 | 1.45E+06 | 8.99E+06 | 2.13E+07 | 1.85E+06 | 1.01E+08 | 14.4 |
| | 1007 | 2.15E+07 | 2.15E+07 | 4.68E+07 | 4.55E+06 | 2.98E+05 | 4.05E+05 | 3.59E+04 | 9.50E+07 | 14.6 |
| | 1059 | 1.76E+07 | 3.65E+06 | 6.29E+07 | 2.01E+06 | 1.14E+07 | 2.60E+07 | 1.49E+06 | 1.25E+08 | 15.2 |
| | 1040 | 2.54E+07 | 9.30E+06 | 6.55E+07 | 9.50E+04 | 8.60E+06 | 1.77E+07 | 1.43E+06 | 1.28E+08 | 15.8 |
| | 1019 | 1.39E+07 | 1.36E+07 | 4.77E+07 | 7.37E+05 | 7.15E+06 | 2.15E+07 | 1.39E+06 | 1.06E+08 | 16.0 |
| | 1053 | 3.22E+07 | 5.37E+06 | 5.94E+07 | 1.42E+07 | 5.54E+05 | 2.03E+07 | 2.65E+06 | 1.35E+08 | 16.2 |
| | 1029 | 1.33E+07 | 1.24E+07 | 5.36E+07 | 5.47E+06 | 2.25E+06 | 1.99E+07 | 1.55E+06 | 1.08E+08 | 16.8 |
| | 1008 | 2.05E+07 | 9.95E+06 | 7.72E+07 | 1.05E+06 | 9.83E+06 | 1.68E+07 | 1.24E+06 | 1.37E+08 | 16.9 |
| | 1028 | 3.23E+07 | 7.13E+06 | 6.40E+07 | 2.14E+06 | 9.60E+06 | 2.65E+07 | 1.88E+06 | 1.44E+08 | 17.3 |
| | 1014 | 1.43E+07 | 5.77E+06 | 5.01E+07 | 1.73E+06 | 4.60E+06 | 2.55E+07 | 2.42E+06 | 1.04E+08 | 17.5 |
| | 1032 | 1.10E+07 | 2.41E+06 | 5.62E+07 | 2.13E+06 | 7.20E+06 | 3.01E+07 | 1.61E+06 | 1.11E+08 | 17.5 |
| | 1065 | 3.52E+07 | 1.29E+07 | 5.47E+07 | 9.50E+04 | 9.44E+04 | 1.27E+05 | 3.59E+04 | 1.03E+08 | 18.0 |
| | 1033 | 3.30E+07 | 4.10E+06 | 6.14E+07 | 2.51E+06 | 3.17E+04 | 4.51E+07 | 1.95E+06 | 1.48E+08 | 18.7 |
| | 1064 | 2.00E+07 | 8.25E+06 | 5.43E+07 | 1.99E+06 | 2.86E+06 | 3.20E+07 | 3.32E+06 | 1.23E+08 | 18.8 |
| | 1009 | 3.47E+07 | 6.75E+06 | 5.75E+07 | 3.13E+06 | 6.68E+06 | 4.21E+07 | 4.10E+06 | 1.55E+08 | 19.5 |
| | 1043 | 1.12E+07 | 3.58E+06 | 6.19E+07 | 2.35E+06 | 2.54E+06 | 3.59E+07 | 2.83E+06 | 1.20E+08 | 19.7 |
| | 1012 | 1.48E+07 | 7.14E+06 | 5.74E+07 | 5.03E+05 | 1.21E+06 | 2.99E+07 | 1.64E+06 | 1.13E+08 | 19.7 |
| | 1060 | 2.42E+07 | 1.21E+07 | 4.80E+07 | 6.16E+05 | 3.17E+04 | 2.38E+07 | 2.09E+06 | 1.11E+08 | 20.1 |
| | 1050 | 4.70E+07 | 2.60E+07 | 3.49E+07 | 9.92E+05 | 1.13E+06 | 2.23E+07 | 4.13E+06 | 1.36E+08 | 21.1 |
| | 1011 | 2.41E+07 | 3.80E+06 | 6.40E+07 | 3.01E+06 | 1.01E+07 | 4.41E+07 | 2.18E+06 | 1.51E+08 | 21.2 |
| | 1000 | 3.02E+07 | 7.00E+06 | 5.49E+07 | 1.90E+05 | 5.37E+06 | 2.72E+07 | 1.74E+06 | 1.27E+08 | 21.7 |
| | 1039 | 1.29E+07 | 1.13E+07 | 4.19E+07 | 1.03E+07 | 9.60E+06 | 3.22E+06 | 2.73E+05 | 8.95E+07 | 22.3 |
| | 1025 | 3.81E+07 | 1.82E+07 | 4.96E+07 | 4.50E+05 | 4.75E+06 | 1.53E+07 | 1.37E+06 | 1.28E+08 | 22.6 |
| | 1013 | 2.65E+07 | 1.33E+07 | 5.60E+07 | 1.60E+06 | 1.26E+06 | 3.55E+07 | 3.75E+06 | 1.38E+08 | 22.7 |
| | 1031 | 2.79E+07 | 3.66E+07 | 3.17E+04 | 3.26E+06 | 4.88E+05 | 1.82E+06 | 3.59E+04 | 7.01E+07 | 23.9 |
| | 1034 | 2.63E+07 | 1.44E+07 | 5.08E+07 | 1.17E+07 | 6.05E+06 | 6.34E+06 | 3.03E+05 | 1.16E+08 | 23.9 |
| | 1057 | 5.95E+07 | 2.84E+06 | 6.96E+07 | 2.94E+07 | 8.37E+06 | 5.69E+07 | 4.63E+06 | 2.31E+08 | 24.1 |
| | 1037 | 5.05E+07 | 1.78E+07 | 5.29E+07 | 3.97E+05 | 3.37E+06 | 1.70E+07 | 1.34E+06 | 1.43E+08 | 24.4 |
| | 1005 | 3.00E+07 | 1.33E+07 | 7.99E+07 | 4.71E+07 | 1.51E+07 | 1.67E+07 | 1.61E+06 | 2.04E+08 | 27.3 |
| | 1045 | 1.96E+07 | 3.62E+07 | 2.04E+07 | 1.70E+07 | 8.27E+04 | 1.62E+06 | 2.20E+05 | 9.51E+07 | 30.0 |
| Non-Secretor | Avg | 2.60E+05 | 7.65E+05 | 7.04E+04 | 2.14E+06 | 6.60E+04 | 1.17E+06 | 2.32E+05 | 4.70E+06 | 0.912 |
| | Stdev | 5.07E+05 | 2.24E+06 | 4.11E+04 | 2.02E+06 | 1.04E+05 | 1.49E+06 | 2.04E+05 | 2.92E+06 | 0.886 |
| Secretor | Avg | 2.50E+07 | 1.15E+07 | 5.30E+07 | 5.66E+06 | 4.78E+06 | 2.15E+07 | 1.74E+06 | 1.23E+08 | 19.290 |
| | Stdev | 1.20E+07 | 8.49E+06 | 1.47E+07 | 9.73E+06 | 4.09E+06 | 1.43E+07 | 1.24E+06 | 3.23E+07 | 4.180 |
