# Supplementary figures and images for: Maternal fucosyltransferase 2 status affects the gut bifidobacterial communities of breastfed infants
Source: Microbiome. 2015 Apr 10;3:13. doi: 10.1186/s40168-015-0071-z (PMC4412032; doi:10.1186/s40168-015-0071-z)

## Slide 1
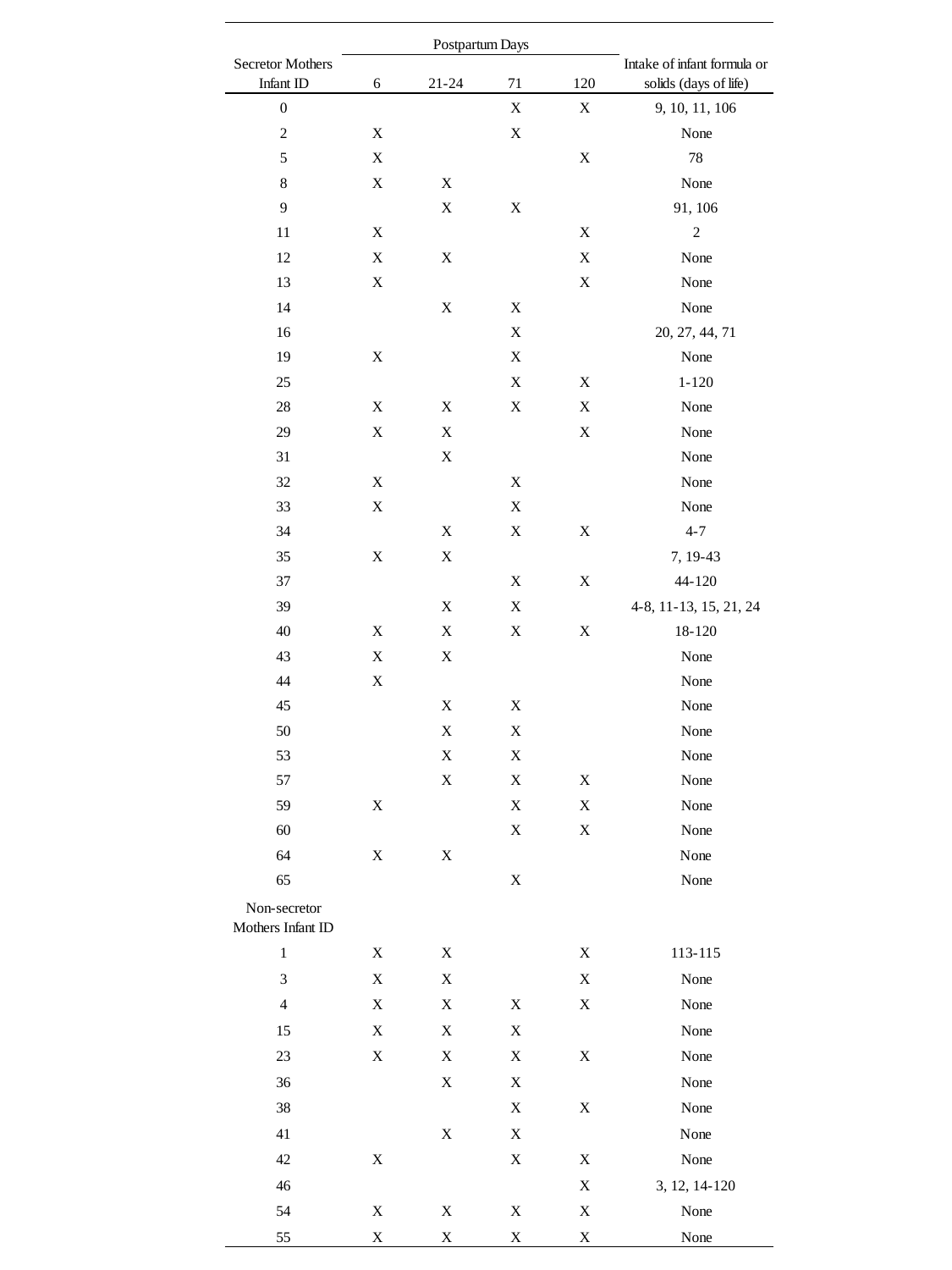

Supplement: Additional file 2: Table S2. — Breast milk and infant fecal sampling from mother-infant dyads per time point. 1Self report by parent who was prompted to answer the question ‘has your infant consumed any solids or infant formula’ with each stool sample. The days of life listed include each day when solids or infant formula intake was reported. [file 40168_2015_71_MOESM2_ESM.pptx]
