# Supplementary material for: Maternal fucosyltransferase 2 status affects the gut bifidobacterial communities of breastfed infants
Source: Microbiome. 2015 Apr 10;3:13. doi: 10.1186/s40168-015-0071-z (PMC4412032; doi:10.1186/s40168-015-0071-z)
Supplement: Additional file 3: Figure S1. — C-section (N = 5) versus vaginal (N = 39) birth over time. Shows composition of the microbiota as determined by marker gene sequencing. [file 40168_2015_71_MOESM3_ESM.pptx]

## Slide 1
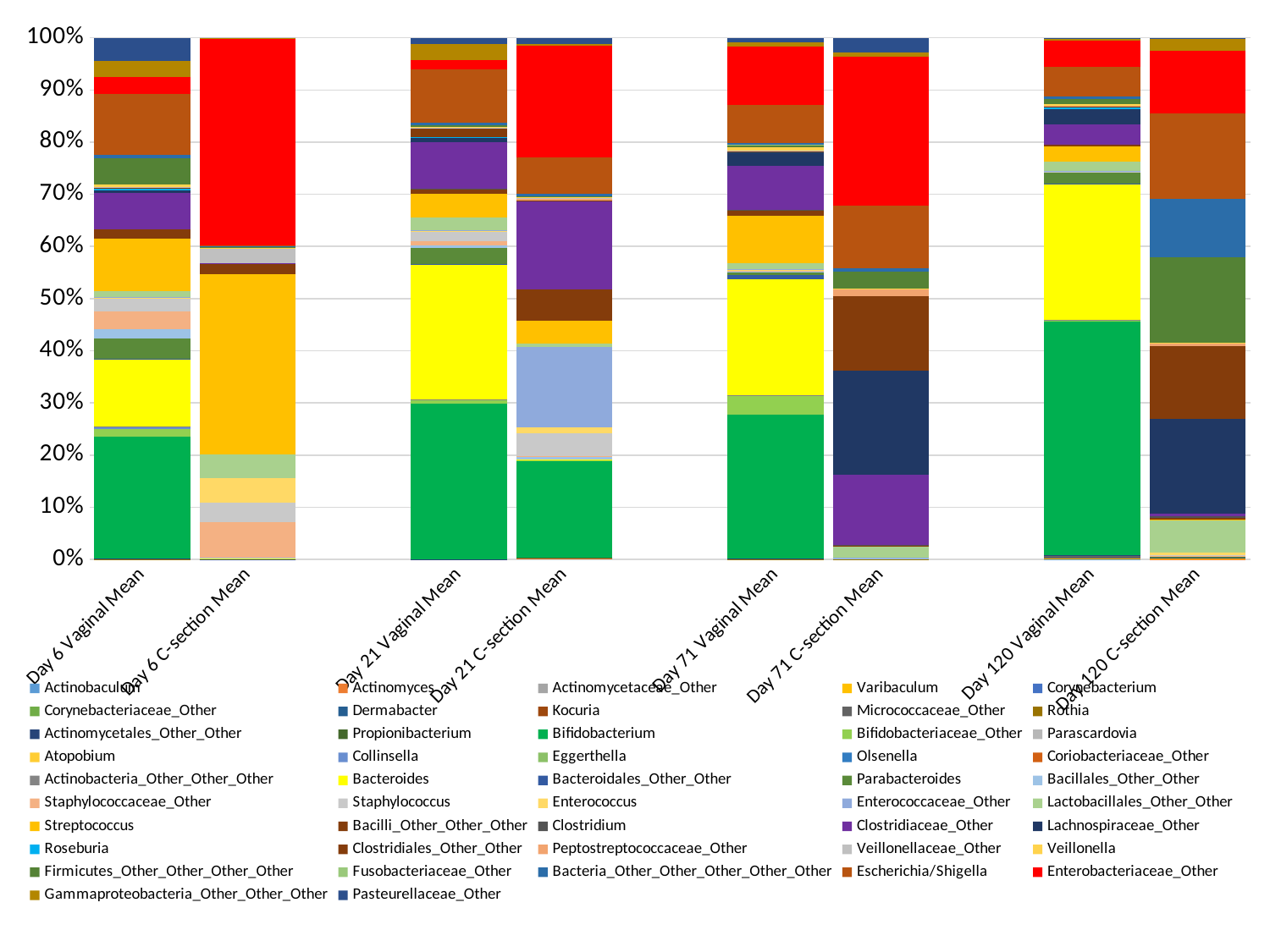

### Chart
| Category | Actinobaculum | Actinomyces | Actinomycetaceae_Other | Varibaculum | Corynebacterium | Corynebacteriaceae_Other | Dermabacter | Kocuria | Micrococcaceae_Other | Rothia | Actinomycetales_Other_Other | Propionibacterium | Bifidobacterium | Bifidobacteriaceae_Other | Parascardovia | Atopobium | Collinsella | Eggerthella | Olsenella | Coriobacteriaceae_Other | Actinobacteria_Other_Other_Other | Bacteroides | Bacteroidales_Other_Other | Parabacteroides | Bacillales_Other_Other | Staphylococcaceae_Other | Staphylococcus | Enterococcus | Enterococcaceae_Other | Lactobacillales_Other_Other | Streptococcus | Bacilli_Other_Other_Other | Clostridium | Clostridiaceae_Other | Lachnospiraceae_Other | Roseburia | Clostridiales_Other_Other | Peptostreptococcaceae_Other | Veillonellaceae_Other | Veillonella | Firmicutes_Other_Other_Other_Other | Fusobacteriaceae_Other | Bacteria_Other_Other_Other_Other_Other | Escherichia/Shigella | Enterobacteriaceae_Other | Gammaproteobacteria_Other_Other_Other | Pasteurellaceae_Other |
|---|---|---|---|---|---|---|---|---|---|---|---|---|---|---|---|---|---|---|---|---|---|---|---|---|---|---|---|---|---|---|---|---|---|---|---|---|---|---|---|---|---|---|---|---|---|---|---|
| Day 6 Vaginal Mean | 0.0 | 1.30434782608696e-05 | 0.0 | 0.0 | 0.0 | 0.0 | 0.0 | 0.0 | 0.0 | 0.000808695652173913 | 0.000269565217391304 | 0.0 | 0.2339 | 0.0144869565217391 | 0.0 | 0.0 | 0.00445217391304348 | 2.60869565217391e-05 | 0.0 | 2.17391304347826e-05 | 7.82608695652174e-05 | 0.128326086956522 | 0.000317391304347826 | 0.0399478260869565 | 0.017904347826087 | 0.0326652173913044 | 0.0242695652173913 | 0.00323478260869565 | 0.000417391304347826 | 0.0121782608695652 | 0.100234782608696 | 0.0164086956521739 | 0.0 | 0.070795652173913 | 0.00468260869565218 | 0.00260869565217391 | 0.00210869565217391 | 0.00117391304347826 | 0.00051304347826087 | 0.00454347826086957 | 0.0502608695652174 | 1.73913043478261e-05 | 0.00529130434782609 | 0.116930434782609 | 0.0328608695652174 | 0.0300695652173913 | 0.0445478260869565 |
| Day 6 C-section Mean | 0.0 | 0.0 | 0.0 | 0.0 | 0.0 | 0.0 | 0.0 | 0.0 | 0.0001 | 0.00025 | 5e-05 | 0.0 | 0.0008 | 5e-05 | 0.0 | 0.0 | 0.0 | 0.0 | 0.0 | 0.0 | 0.0 | 0.0005 | 0.0 | 0.0 | 0.0011 | 0.06855 | 0.0378 | 0.04615 | 0.0 | 0.04575 | 0.3461 | 0.02005 | 0.0 | 0.00075 | 0.0 | 0.0 | 0.0 | 0.0 | 0.02885 | 0.0003 | 0.0008 | 0.0 | 0.0028 | 0.00045 | 0.39785 | 0.00015 | 5e-05 |
| | None | None | None | None | None | None | None | None | None | None | None | None | None | None | None | None | None | None | None | None | None | None | None | None | None | None | None | None | None | None | None | None | None | None | None | None | None | None | None | None | None | None | None | None | None | None | None |
| Day 21 Vaginal Mean | 4.34782608695652e-05 | 0.00011304347826087 | 0.0 | 1.30434782608696e-05 | 3.04347826086957e-05 | 4.34782608695652e-06 | 0.0 | 0.0 | 4.34782608695652e-06 | 0.000430434782608696 | 0.000152173913043478 | 1.73913043478261e-05 | 0.295569565217391 | 0.00718260869565218 | 0.0 | 5.21739130434783e-05 | 0.00041304347826087 | 0.00011304347826087 | 0.0 | 0.000108695652173913 | 0.000952173913043478 | 0.255473913043478 | 0.00124782608695652 | 0.0313608695652174 | 0.00360869565217391 | 0.00909130434782608 | 0.0179521739130435 | 0.000652173913043478 | 0.00233478260869565 | 0.0241478260869565 | 0.0446304347826087 | 0.00867391304347826 | 0.0017695652173913 | 0.0876434782608695 | 0.00815217391304348 | 0.001 | 0.0176869565217391 | 0.00038695652173913 | 0.000591304347826087 | 0.00155217391304348 | 0.00333913043478261 | 2.60869565217391e-05 | 0.00381304347826087 | 0.101934782608696 | 0.0187304347826087 | 0.0300782608695652 | 0.0114869565217391 |
| Day 21 C-section Mean | 0.0 | 0.00205 | 0.0 | 0.0 | 2.5e-05 | 0.0 | 2.5e-05 | 0.0 | 0.0 | 0.000725 | 0.00015 | 0.000175 | 0.184625 | 0.003225 | 0.0 | 0.0 | 0.0 | 0.0 | 0.0 | 0.0 | 5e-05 | 0.000225 | 0.0 | 0.0 | 0.004175 | 0.003 | 0.04265 | 0.011375 | 0.154625 | 0.0058 | 0.044125 | 0.060025 | 0.0 | 0.17 | 2.5e-05 | 0.0 | 0.0003 | 0.0047 | 2.5e-05 | 0.001625 | 0.001775 | 0.0 | 0.0044 | 0.069075 | 0.2147 | 0.003525 | 0.011125 |
| | None | None | None | None | None | None | None | None | None | None | None | None | None | None | None | None | None | None | None | None | None | None | None | None | None | None | None | None | None | None | None | None | None | None | None | None | None | None | None | None | None | None | None | None | None | None | None |
| Day 71 Vaginal Mean | 0.0 | 0.000307692307692308 | 7.69230769230769e-06 | 1.15384615384615e-05 | 1.53846153846154e-05 | 3.84615384615385e-06 | 3.84615384615385e-06 | 3.84615384615385e-06 | 7.69230769230769e-06 | 0.000369230769230769 | 0.000176923076923077 | 0.0 | 0.274673076923077 | 0.0370538461538462 | 3.84615384615385e-06 | 0.000142307692307692 | 0.000338461538461538 | 7.69230769230769e-05 | 0.0 | 0.0 | 0.000107692307692308 | 0.219973076923077 | 0.00811153846153846 | 0.00687307692307692 | 0.000592307692307692 | 0.00155 | 0.00146153846153846 | 0.0002 | 0.001 | 0.0119230769230769 | 0.0907269230769231 | 0.0111807692307692 | 6.92307692307692e-05 | 0.0837961538461539 | 0.0245 | 5.76923076923077e-05 | 0.00331538461538462 | 0.000446153846153846 | 0.000657692307692308 | 0.00560384615384615 | 0.0046 | 0.000542307692307692 | 0.00353461538461538 | 0.0731423076923077 | 0.1101 | 0.00922692307692308 | 0.00785384615384615 |
| Day 71 C-section Mean | 0.0 | 7.5e-05 | 0.0 | 0.0 | 5e-05 | 0.0 | 0.0 | 0.0 | 0.0 | 0.0007 | 0.0001 | 0.0 | 0.000575 | 5e-05 | 0.0 | 0.0 | 0.0 | 5e-05 | 0.0 | 0.0 | 0.0 | 0.0003 | 0.0 | 0.0 | 0.000525 | 0.0003 | 0.000725 | 0.0004 | 5e-05 | 0.019575 | 0.00195 | 0.001175 | 0.0016 | 0.133325 | 0.19795 | 0.0 | 0.141475 | 0.01295 | 0.0 | 0.000625 | 0.03315 | 0.0 | 0.005625 | 0.119975 | 0.2831 | 0.007875 | 0.027725 |
| | None | None | None | None | None | None | None | None | None | None | None | None | None | None | None | None | None | None | None | None | None | None | None | None | None | None | None | None | None | None | None | None | None | None | None | None | None | None | None | None | None | None | None | None | None | None | None |
| Day 120 Vaginal Mean | 9.52380952380953e-06 | 0.000966666666666667 | 4.76190476190476e-06 | 0.00115238095238095 | 4.76190476190476e-06 | 0.0 | 1.90476190476191e-05 | 9.52380952380953e-06 | 0.00480952380952381 | 0.00104761904761905 | 0.000566666666666667 | 0.0 | 0.441871428571429 | 0.00311904761904762 | 4.76190476190476e-06 | 3.33333333333333e-05 | 3.33333333333333e-05 | 9.52380952380953e-05 | 0.0 | 9.52380952380953e-06 | 0.000133333333333333 | 0.258038095238095 | 0.000614285714285714 | 0.0220809523809524 | 0.0002 | 0.000695238095238095 | 0.000380952380952381 | 0.000795238095238095 | 0.000790476190476191 | 0.0177190476190476 | 0.0285380952380952 | 0.00357142857142857 | 9.52380952380953e-06 | 0.0384619047619048 | 0.0281285714285714 | 0.0027 | 0.0038 | 0.000204761904761905 | 5.23809523809524e-05 | 0.00232380952380952 | 0.0109952380952381 | 1.90476190476191e-05 | 0.00395714285714286 | 0.0559619047619048 | 0.0502571428571429 | 0.00466190476190476 | 0.000552380952380952 |
| Day 120 C-section Mean | 0.0 | 0.0004 | 3.33333333333333e-05 | 0.00206666666666667 | 0.0 | 0.0 | 0.0 | 0.0 | 0.0 | 0.0002 | 0.000966666666666667 | 0.0 | 0.0006 | 0.0 | 0.0 | 0.0004 | 0.0 | 0.0001 | 0.000433333333333333 | 0.0 | 0.0 | 0.000266666666666667 | 0.0 | 6.66666666666667e-05 | 0.000433333333333333 | 0.000166666666666667 | 0.0017 | 0.0046 | 0.0 | 0.0621666666666667 | 0.00166666666666667 | 0.00243333333333333 | 0.0036 | 0.00516666666666667 | 0.1788 | 0.0 | 0.139366666666667 | 0.00523333333333334 | 0.0 | 0.0007 | 0.162933333333333 | 0.0 | 0.110266666666667 | 0.161733333333333 | 0.120033333333333 | 0.0231333333333333 | 0.0008 |
