# Supplementary material for: Maternal fucosyltransferase 2 status affects the gut bifidobacterial communities of breastfed infants
Source: Microbiome. 2015 Apr 10;3:13. doi: 10.1186/s40168-015-0071-z (PMC4412032; doi:10.1186/s40168-015-0071-z)
Supplement: Additional file 4: Figure S2. — Example growth curves of bifidobacterial growth on 2′-fucosyllactose. One representative growth curve for each level of OD (high ≥ 0.8, medium = 0.4 to 0.8, low ≤ 0.4) is shown here. Data in Figure 13 shows the breakdown of all unique isolates by this assignment method. [file 40168_2015_71_MOESM4_ESM.pptx]

## Slide 1
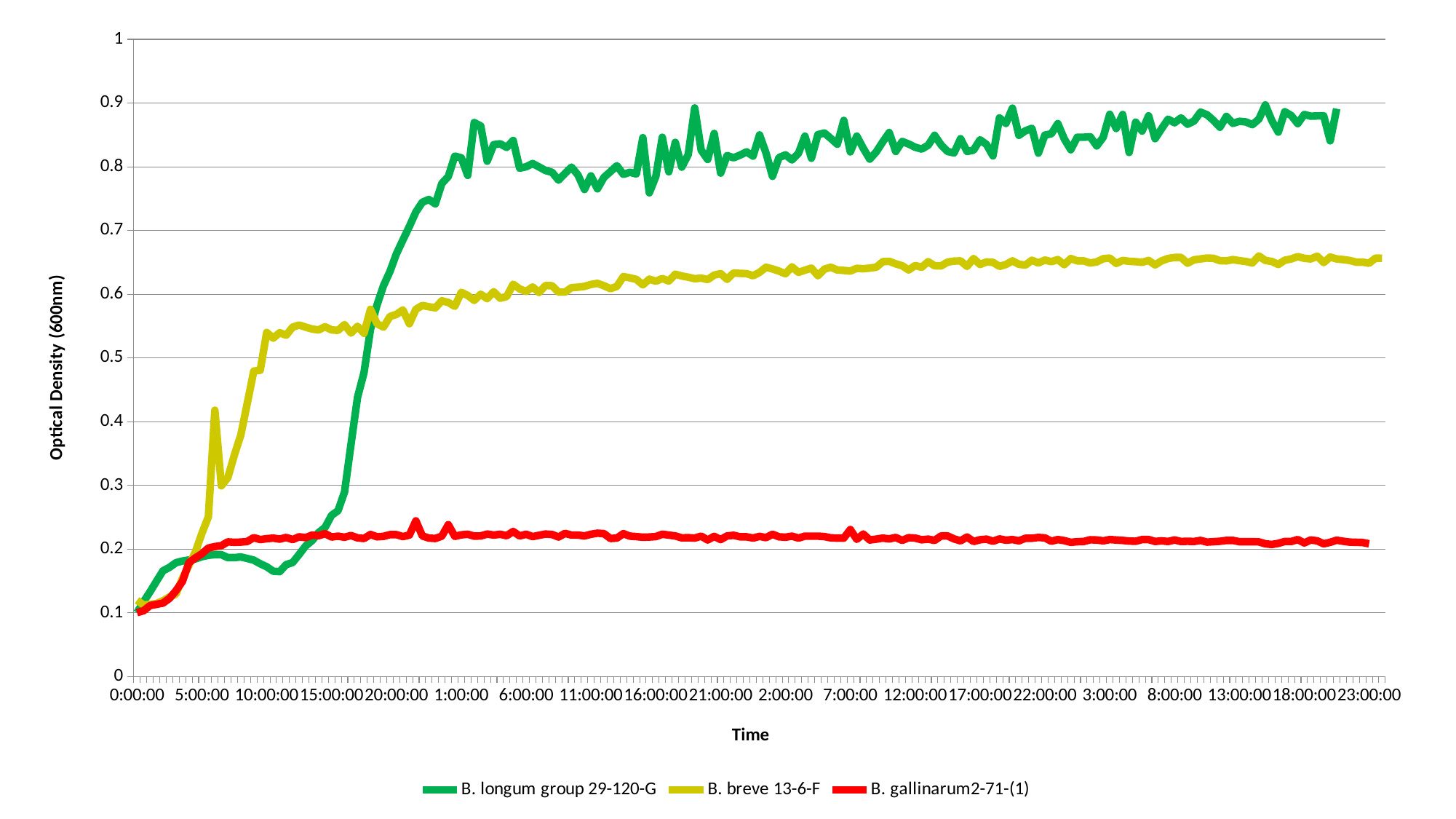

### Chart
| Category | B. longum group 29-120-G | B. breve 13-6-F | B. gallinarum2-71-(1) |
|---|---|---|---|
| 0 | 0.100333333333333 | 0.119666666666667 | 0.100333333333333 |
| 2.0833333333333301E-2 | 0.117333333333333 | 0.112333333333333 | 0.103333333333333 |
| 4.1666666666666699E-2 | 0.133 | 0.113333333333333 | 0.111333333333333 |
| 6.25E-2 | 0.149666666666667 | 0.114666666666667 | 0.113333333333333 |
| 8.3333333333333301E-2 | 0.166 | 0.119 | 0.115333333333333 |
| 0.104166666666667 | 0.171333333333333 | 0.125 | 0.122666666666667 |
| 0.125 | 0.178666666666667 | 0.129333333333333 | 0.134666666666667 |
| 0.14583333333333301 | 0.181333333333333 | 0.154 | 0.149333333333333 |
| 0.16666666666666699 | 0.183 | 0.174333333333333 | 0.178666666666667 |
| 0.1875 | 0.184666666666667 | 0.195666666666667 | 0.186333333333333 |
| 0.20833333333333301 | 0.188 | 0.224666666666667 | 0.192666666666667 |
| 0.22916666666666699 | 0.190333333333333 | 0.250666666666667 | 0.201666666666667 |
| 0.25 | 0.191333333333333 | 0.418 | 0.204 |
| 0.27083333333333298 | 0.191333333333333 | 0.299333333333333 | 0.205333333333333 |
| 0.29166666666666702 | 0.186666666666667 | 0.312 | 0.211333333333333 |
| 0.3125 | 0.186666666666667 | 0.347333333333333 | 0.210666666666667 |
| 0.33333333333333298 | 0.187666666666667 | 0.379 | 0.211 |
| 0.35416666666666702 | 0.185333333333333 | 0.428666666666667 | 0.212 |
| 0.375 | 0.182666666666667 | 0.479333333333333 | 0.218 |
| 0.39583333333333298 | 0.177 | 0.480666666666667 | 0.215 |
| 0.41666666666666702 | 0.172333333333333 | 0.54 | 0.216333333333333 |
| 0.4375 | 0.165333333333333 | 0.531 | 0.217333333333333 |
| 0.45833333333333298 | 0.164666666666667 | 0.539666666666667 | 0.215666666666667 |
| 0.47916666666666702 | 0.175666666666667 | 0.535666666666667 | 0.218333333333333 |
| 0.5 | 0.179 | 0.548333333333333 | 0.215 |
| 0.52083333333333304 | 0.191666666666667 | 0.551666666666667 | 0.219333333333333 |
| 0.54166666666666696 | 0.205 | 0.548333333333333 | 0.218 |
| 0.5625 | 0.213333333333333 | 0.545333333333333 | 0.222 |
| 0.58333333333333304 | 0.226 | 0.544 | 0.221 |
| 0.60416666666666696 | 0.234 | 0.549 | 0.224333333333333 |
| 0.625 | 0.253 | 0.544 | 0.219 |
| 0.64583333333333304 | 0.260333333333333 | 0.543333333333333 | 0.220333333333333 |
| 0.66666666666666696 | 0.289666666666667 | 0.552333333333333 | 0.218666666666667 |
| 0.6875 | 0.364666666666667 | 0.539 | 0.221333333333333 |
| 0.70833333333333304 | 0.438 | 0.549666666666667 | 0.217666666666667 |
| 0.72916666666666696 | 0.477 | 0.538666666666667 | 0.216666666666667 |
| 0.75 | 0.545 | 0.576333333333333 | 0.223 |
| 0.77083333333333304 | 0.583 | 0.553666666666667 | 0.219333333333333 |
| 0.79166666666666696 | 0.613333333333333 | 0.548333333333333 | 0.22 |
| 0.8125 | 0.635666666666667 | 0.565 | 0.222666666666667 |
| 0.83333333333333304 | 0.663333333333333 | 0.568333333333333 | 0.222666666666667 |
| 0.85416666666666696 | 0.685 | 0.575333333333333 | 0.219666666666667 |
| 0.875 | 0.706333333333333 | 0.553666666666667 | 0.222 |
| 0.89583333333333304 | 0.729 | 0.576333333333333 | 0.244333333333333 |
| 0.91666666666666696 | 0.744333333333333 | 0.582333333333333 | 0.220666666666667 |
| 0.9375 | 0.748666666666667 | 0.580333333333333 | 0.217333333333333 |
| 0.95833333333333304 | 0.741666666666667 | 0.578666666666667 | 0.216666666666667 |
| 0.97916666666666696 | 0.773666666666667 | 0.589666666666667 | 0.220333333333333 |
| 1 | 0.784666666666667 | 0.587 | 0.238333333333333 |
| 1.020833333333333 | 0.816666666666667 | 0.581 | 0.22 |
| 1.041666666666667 | 0.814 | 0.603 | 0.222333333333333 |
| 1.0625 | 0.786333333333333 | 0.598 | 0.223333333333333 |
| 1.083333333333333 | 0.869333333333333 | 0.590333333333333 | 0.220333333333333 |
| 1.104166666666667 | 0.864 | 0.6 | 0.220666666666667 |
| 1.125 | 0.808666666666667 | 0.593 | 0.223666666666667 |
| 1.145833333333333 | 0.834666666666667 | 0.604 | 0.222 |
| 1.166666666666667 | 0.836 | 0.593666666666667 | 0.223333333333333 |
| 1.1875 | 0.830333333333333 | 0.596333333333333 | 0.221 |
| 1.208333333333333 | 0.841333333333333 | 0.615666666666667 | 0.227666666666667 |
| 1.229166666666667 | 0.797666666666667 | 0.608333333333333 | 0.220666666666667 |
| 1.25 | 0.8 | 0.604333333333333 | 0.223333333333333 |
| 1.270833333333333 | 0.805 | 0.611333333333333 | 0.219666666666667 |
| 1.291666666666667 | 0.799666666666667 | 0.602666666666666 | 0.221666666666667 |
| 1.3125 | 0.794 | 0.613666666666667 | 0.223666666666667 |
| 1.333333333333333 | 0.791333333333333 | 0.613333333333333 | 0.223 |
| 1.354166666666667 | 0.779 | 0.603333333333333 | 0.219 |
| 1.375 | 0.789666666666667 | 0.603333333333333 | 0.224666666666667 |
| 1.395833333333333 | 0.799333333333333 | 0.610333333333333 | 0.222 |
| 1.416666666666667 | 0.787 | 0.611 | 0.222 |
| 1.4375 | 0.764333333333333 | 0.612333333333333 | 0.220666666666667 |
| 1.458333333333333 | 0.785666666666667 | 0.615333333333333 | 0.223333333333333 |
| 1.479166666666667 | 0.765333333333333 | 0.617 | 0.225 |
| 1.5 | 0.783333333333333 | 0.613333333333333 | 0.224333333333333 |
| 1.520833333333333 | 0.792333333333333 | 0.608666666666667 | 0.216666666666667 |
| 1.541666666666667 | 0.801666666666667 | 0.612333333333333 | 0.217333333333333 |
| 1.5625 | 0.788 | 0.627666666666667 | 0.224333333333333 |
| 1.583333333333333 | 0.791333333333333 | 0.625666666666667 | 0.220333333333333 |
| 1.604166666666667 | 0.788666666666667 | 0.623333333333333 | 0.219666666666667 |
| 1.625 | 0.845666666666667 | 0.614666666666667 | 0.218666666666667 |
| 1.645833333333333 | 0.759 | 0.623666666666667 | 0.219 |
| 1.666666666666667 | 0.784666666666667 | 0.620333333333333 | 0.219666666666667 |
| 1.6875 | 0.846333333333333 | 0.624666666666667 | 0.223333333333333 |
| 1.708333333333333 | 0.792 | 0.620666666666666 | 0.222 |
| 1.729166666666667 | 0.838333333333333 | 0.631333333333333 | 0.220666666666667 |
| 1.75 | 0.799333333333333 | 0.628666666666667 | 0.217666666666667 |
| 1.770833333333333 | 0.819 | 0.626666666666666 | 0.218 |
| 1.791666666666667 | 0.892333333333333 | 0.624333333333333 | 0.217333333333333 |
| 1.8125 | 0.826 | 0.625333333333333 | 0.220333333333333 |
| 1.833333333333333 | 0.811333333333333 | 0.623 | 0.214333333333333 |
| 1.854166666666667 | 0.852333333333333 | 0.63 | 0.22 |
| 1.875 | 0.79 | 0.632333333333333 | 0.215 |
| 1.895833333333333 | 0.817666666666667 | 0.623333333333333 | 0.220666666666667 |
| 1.916666666666667 | 0.814 | 0.633333333333333 | 0.221666666666667 |
| 1.9375 | 0.818333333333333 | 0.632666666666667 | 0.219333333333333 |
| 1.958333333333333 | 0.823333333333333 | 0.632333333333333 | 0.219333333333333 |
| 1.979166666666667 | 0.816666666666667 | 0.629 | 0.217333333333333 |
| 2 | 0.85 | 0.634333333333333 | 0.22 |
| 2.020833333333333 | 0.821666666666667 | 0.642333333333333 | 0.218 |
| 2.041666666666667 | 0.785 | 0.639666666666667 | 0.223333333333333 |
| 2.0625 | 0.814333333333333 | 0.636333333333333 | 0.219333333333333 |
| 2.0833333333333339 | 0.818666666666667 | 0.632 | 0.218666666666667 |
| 2.104166666666667 | 0.810666666666667 | 0.643 | 0.220333333333333 |
| 2.125 | 0.821 | 0.634333333333333 | 0.217333333333333 |
| 2.145833333333333 | 0.848 | 0.637666666666667 | 0.220333333333333 |
| 2.1666666666666661 | 0.813333333333333 | 0.641 | 0.220333333333333 |
| 2.1875000000000009 | 0.850333333333333 | 0.629 | 0.220333333333333 |
| 2.2083333333333339 | 0.853 | 0.639333333333333 | 0.219666666666667 |
| 2.229166666666667 | 0.844333333333333 | 0.642333333333333 | 0.217666666666667 |
| 2.25 | 0.835333333333333 | 0.638 | 0.217333333333333 |
| 2.270833333333333 | 0.872666666666667 | 0.637333333333333 | 0.217333333333333 |
| 2.2916666666666661 | 0.823333333333333 | 0.636333333333333 | 0.230666666666667 |
| 2.312499999999996 | 0.848 | 0.640666666666667 | 0.215333333333333 |
| 2.333333333333333 | 0.829 | 0.64 | 0.223666666666667 |
| 2.3541666666666661 | 0.812 | 0.641 | 0.214333333333333 |
| 2.3749999999999991 | 0.823333333333333 | 0.642333333333333 | 0.215666666666667 |
| 2.3958333333333321 | 0.839 | 0.651 | 0.217333333333333 |
| 2.4166666666666621 | 0.853666666666667 | 0.651666666666667 | 0.216 |
| 2.4375 | 0.824 | 0.647666666666667 | 0.218333333333333 |
| 2.458333333333333 | 0.839666666666667 | 0.644666666666667 | 0.213666666666667 |
| 2.4791666666666661 | 0.835666666666667 | 0.638 | 0.218 |
| 2.5 | 0.830666666666667 | 0.645 | 0.217333333333333 |
| 2.520833333333333 | 0.827666666666667 | 0.642333333333333 | 0.214666666666667 |
| 2.541666666666667 | 0.833666666666667 | 0.651 | 0.215666666666667 |
| 2.5625 | 0.849333333333333 | 0.644666666666667 | 0.213666666666667 |
| 2.5833333333333339 | 0.834333333333333 | 0.644333333333333 | 0.220666666666667 |
| 2.604166666666667 | 0.824 | 0.650333333333333 | 0.220666666666667 |
| 2.625 | 0.821666666666667 | 0.652 | 0.216 |
| 2.645833333333333 | 0.844 | 0.652333333333333 | 0.213 |
| 2.6666666666666661 | 0.824 | 0.643666666666667 | 0.219 |
| 2.6875000000000009 | 0.826 | 0.656 | 0.212 |
| 2.7083333333333339 | 0.842333333333333 | 0.646666666666667 | 0.214666666666667 |
| 2.729166666666667 | 0.835 | 0.650666666666667 | 0.215666666666667 |
| 2.75 | 0.817 | 0.65 | 0.212333333333333 |
| 2.770833333333333 | 0.876666666666667 | 0.643666666666667 | 0.216 |
| 2.7916666666666661 | 0.868 | 0.646666666666667 | 0.214 |
| 2.812499999999996 | 0.892 | 0.652333333333333 | 0.215 |
| 2.833333333333333 | 0.849333333333333 | 0.647 | 0.213 |
| 2.8541666666666661 | 0.856333333333333 | 0.645666666666667 | 0.217 |
| 2.8749999999999991 | 0.860333333333333 | 0.653333333333333 | 0.217 |
| 2.8958333333333321 | 0.821333333333333 | 0.649 | 0.218333333333333 |
| 2.9166666666666621 | 0.849666666666667 | 0.653666666666667 | 0.217666666666667 |
| 2.9375 | 0.851666666666667 | 0.651 | 0.212333333333333 |
| 2.958333333333333 | 0.867666666666667 | 0.654333333333333 | 0.215 |
| 2.9791666666666661 | 0.843666666666667 | 0.646333333333333 | 0.213333333333333 |
| 3 | 0.826666666666667 | 0.656 | 0.210666666666667 |
| 3.020833333333333 | 0.846333333333333 | 0.652333333333333 | 0.211666666666667 |
| 3.041666666666667 | 0.846333333333333 | 0.652333333333333 | 0.212 |
| 3.0625 | 0.847 | 0.649 | 0.214666666666667 |
| 3.0833333333333339 | 0.832666666666667 | 0.650666666666667 | 0.214333333333333 |
| 3.104166666666667 | 0.846333333333333 | 0.655666666666667 | 0.213 |
| 3.125 | 0.882333333333333 | 0.656666666666667 | 0.215 |
| 3.145833333333333 | 0.86 | 0.648333333333333 | 0.214333333333333 |
| 3.1666666666666661 | 0.882 | 0.653 | 0.213666666666667 |
| 3.1875000000000009 | 0.822333333333333 | 0.651666666666667 | 0.212666666666667 |
| 3.2083333333333339 | 0.87 | 0.651 | 0.212333333333333 |
| 3.229166666666667 | 0.856 | 0.65 | 0.215 |
| 3.25 | 0.88 | 0.653 | 0.215 |
| 3.270833333333333 | 0.844 | 0.646 | 0.212 |
| 3.2916666666666661 | 0.859666666666667 | 0.652333333333333 | 0.213 |
| 3.312499999999996 | 0.874666666666667 | 0.656 | 0.212 |
| 3.333333333333333 | 0.869 | 0.657666666666667 | 0.214333333333333 |
| 3.3541666666666661 | 0.876666666666667 | 0.657666666666667 | 0.212 |
| 3.3749999999999991 | 0.866666666666667 | 0.648666666666667 | 0.212333333333333 |
| 3.3958333333333321 | 0.871666666666667 | 0.654 | 0.212 |
| 3.4166666666666621 | 0.885666666666667 | 0.655333333333333 | 0.213666666666667 |
| 3.4375 | 0.881666666666667 | 0.656666666666667 | 0.211 |
| 3.458333333333333 | 0.872666666666667 | 0.656333333333333 | 0.211666666666667 |
| 3.4791666666666661 | 0.862 | 0.652666666666667 | 0.212333333333333 |
| 3.5 | 0.879 | 0.652333333333333 | 0.213666666666667 |
| 3.520833333333333 | 0.868 | 0.654333333333333 | 0.213666666666667 |
| 3.541666666666667 | 0.871333333333333 | 0.652666666666667 | 0.211666666666667 |
| 3.5625 | 0.870333333333333 | 0.651333333333333 | 0.211666666666667 |
| 3.5833333333333339 | 0.866 | 0.649 | 0.211666666666667 |
| 3.604166666666667 | 0.874666666666667 | 0.66 | 0.211333333333333 |
| 3.625 | 0.897 | 0.653 | 0.208333333333333 |
| 3.645833333333333 | 0.872333333333333 | 0.651333333333333 | 0.207333333333333 |
| 3.6666666666666661 | 0.854333333333333 | 0.646666666666667 | 0.209 |
| 3.6875000000000009 | 0.886333333333333 | 0.653333333333333 | 0.212 |
| 3.7083333333333339 | 0.880333333333333 | 0.655333333333333 | 0.212 |
| 3.729166666666667 | 0.867666666666667 | 0.659 | 0.215 |
| 3.75 | 0.882 | 0.656333333333333 | 0.209666666666667 |
| 3.770833333333333 | 0.879333333333333 | 0.655333333333333 | 0.214333333333333 |
| 3.7916666666666661 | 0.879666666666667 | 0.659666666666667 | 0.213 |
| 3.812499999999996 | 0.88 | 0.649666666666667 | 0.208333333333333 |
| 3.833333333333333 | 0.840666666666667 | 0.658333333333333 | 0.210666666666667 |
| 3.8541666666666661 | 0.891 | 0.655333333333333 | 0.214 |
| 3.8749999999999991 | None | 0.654333333333333 | 0.212333333333333 |
| 3.8958333333333321 | None | 0.653 | 0.211 |
| 3.9166666666666621 | None | 0.650333333333333 | 0.210666666666667 |
| 3.9375 | None | 0.650333333333333 | 0.210333333333333 |
| 3.958333333333333 | None | 0.648666666666667 | 0.208333333333333 |
| 3.9791666666666661 | None | 0.656666666666667 | None |
| 4 | None | 0.656333333333333 | None |
