# Supplementary material for: Maternal fucosyltransferase 2 status affects the gut bifidobacterial communities of breastfed infants
Source: Microbiome. 2015 Apr 10;3:13. doi: 10.1186/s40168-015-0071-z (PMC4412032; doi:10.1186/s40168-015-0071-z)
Supplement: Additional file 5: Table S3. — BIO-ENV analysis. Taxa used for evaluation of importance to overall variation between microbial communities of samples, along with iterative results adding in the next most important group and respective Rho statistics for each iteration. [file 40168_2015_71_MOESM5_ESM.pptx]

## Slide 1
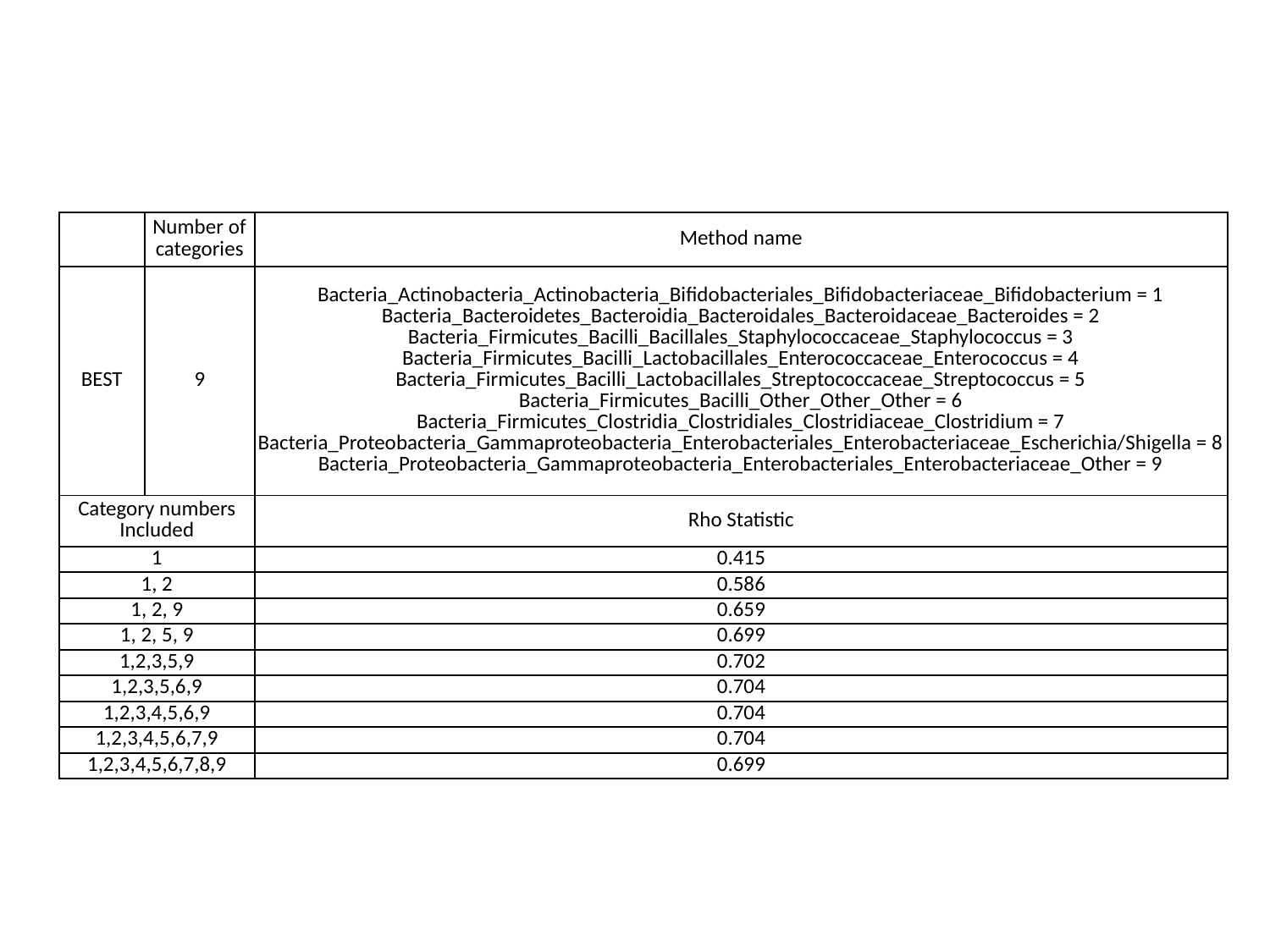

| | Number of categories | Method name |
| --- | --- | --- |
| BEST | 9 | Bacteria\_Actinobacteria\_Actinobacteria\_Bifidobacteriales\_Bifidobacteriaceae\_Bifidobacterium = 1 Bacteria\_Bacteroidetes\_Bacteroidia\_Bacteroidales\_Bacteroidaceae\_Bacteroides = 2 Bacteria\_Firmicutes\_Bacilli\_Bacillales\_Staphylococcaceae\_Staphylococcus = 3 Bacteria\_Firmicutes\_Bacilli\_Lactobacillales\_Enterococcaceae\_Enterococcus = 4 Bacteria\_Firmicutes\_Bacilli\_Lactobacillales\_Streptococcaceae\_Streptococcus = 5 Bacteria\_Firmicutes\_Bacilli\_Other\_Other\_Other = 6 Bacteria\_Firmicutes\_Clostridia\_Clostridiales\_Clostridiaceae\_Clostridium = 7 Bacteria\_Proteobacteria\_Gammaproteobacteria\_Enterobacteriales\_Enterobacteriaceae\_Escherichia/Shigella = 8 Bacteria\_Proteobacteria\_Gammaproteobacteria\_Enterobacteriales\_Enterobacteriaceae\_Other = 9 |
| Category numbers Included | | Rho Statistic |
| 1 | | 0.415 |
| 1, 2 | | 0.586 |
| 1, 2, 9 | | 0.659 |
| 1, 2, 5, 9 | | 0.699 |
| 1,2,3,5,9 | | 0.702 |
| 1,2,3,5,6,9 | | 0.704 |
| 1,2,3,4,5,6,9 | | 0.704 |
| 1,2,3,4,5,6,7,9 | | 0.704 |
| 1,2,3,4,5,6,7,8,9 | | 0.699 |
