# Supplementary material for: Maternal fucosyltransferase 2 status affects the gut bifidobacterial communities of breastfed infants
Source: Microbiome. 2015 Apr 10;3:13. doi: 10.1186/s40168-015-0071-z (PMC4412032; doi:10.1186/s40168-015-0071-z)
Supplement: Additional file 6: Figure S3. — PCoA plots of the NGS data. Colored by days after birth (left), assigned group (center) and mother’s secretor phenotype (right). [file 40168_2015_71_MOESM6_ESM.pptx]

## Slide 1
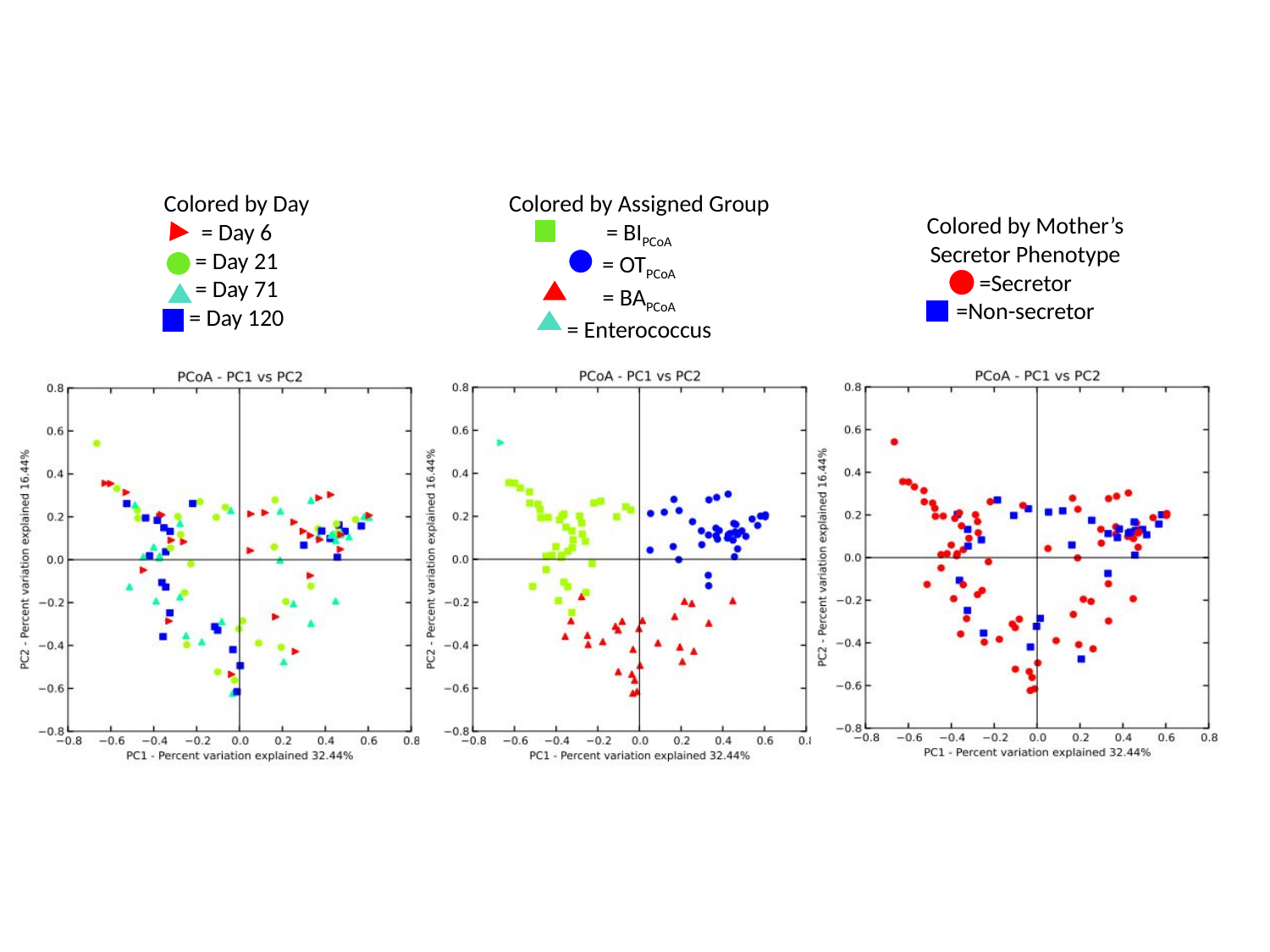

Colored by Day
= Day 6
= Day 21
= Day 71
= Day 120
Colored by Assigned Group
= BIPCoA
= OTPCoA
= BAPCoA
= Enterococcus
Colored by Mother’s
Secretor Phenotype
=Secretor
=Non-secretor
