# Supplementary material for: Maternal fucosyltransferase 2 status affects the gut bifidobacterial communities of breastfed infants
Source: Microbiome. 2015 Apr 10;3:13. doi: 10.1186/s40168-015-0071-z (PMC4412032; doi:10.1186/s40168-015-0071-z)
Supplement: Additional file 7: Figure S4. — PCoA plots of the NGS data. Colored by the abundance of Bifidobacterium (left), Bifidobacteria from qPCR data (center) and Bacteroides (right). Colors represent a spectrum of abundance, with blue being high and red being low. [file 40168_2015_71_MOESM7_ESM.pptx]

## Slide 1
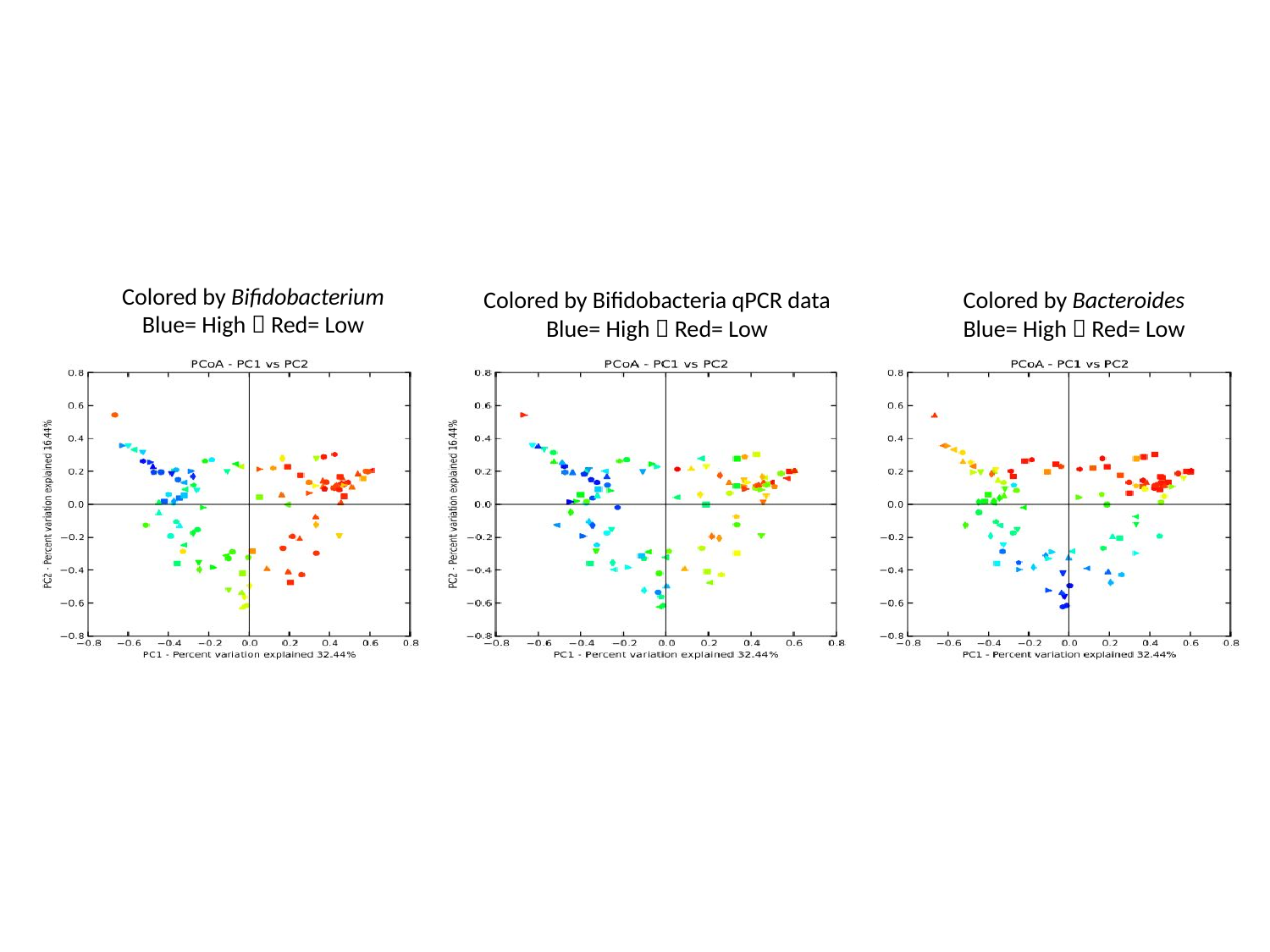

Colored by Bifidobacterium
Blue= High  Red= Low
Colored by Bifidobacteria qPCR data
Blue= High  Red= Low
Colored by Bacteroides
Blue= High  Red= Low
