# Supplementary material for: Maternal fucosyltransferase 2 status affects the gut bifidobacterial communities of breastfed infants
Source: Microbiome. 2015 Apr 10;3:13. doi: 10.1186/s40168-015-0071-z (PMC4412032; doi:10.1186/s40168-015-0071-z)
Supplement: Additional file 8: Figure S5. — PCoA plots of the NGS data. Colored by the abundance of ‘Clostridiaceae_other’ (top left), Escherichia/Shigella (top right), Veillonella (bottom left), and Streptococcus (bottom right). Colors represent a spectrum of abundance, with blue being high and red being low. [file 40168_2015_71_MOESM8_ESM.pptx]

## Slide 1
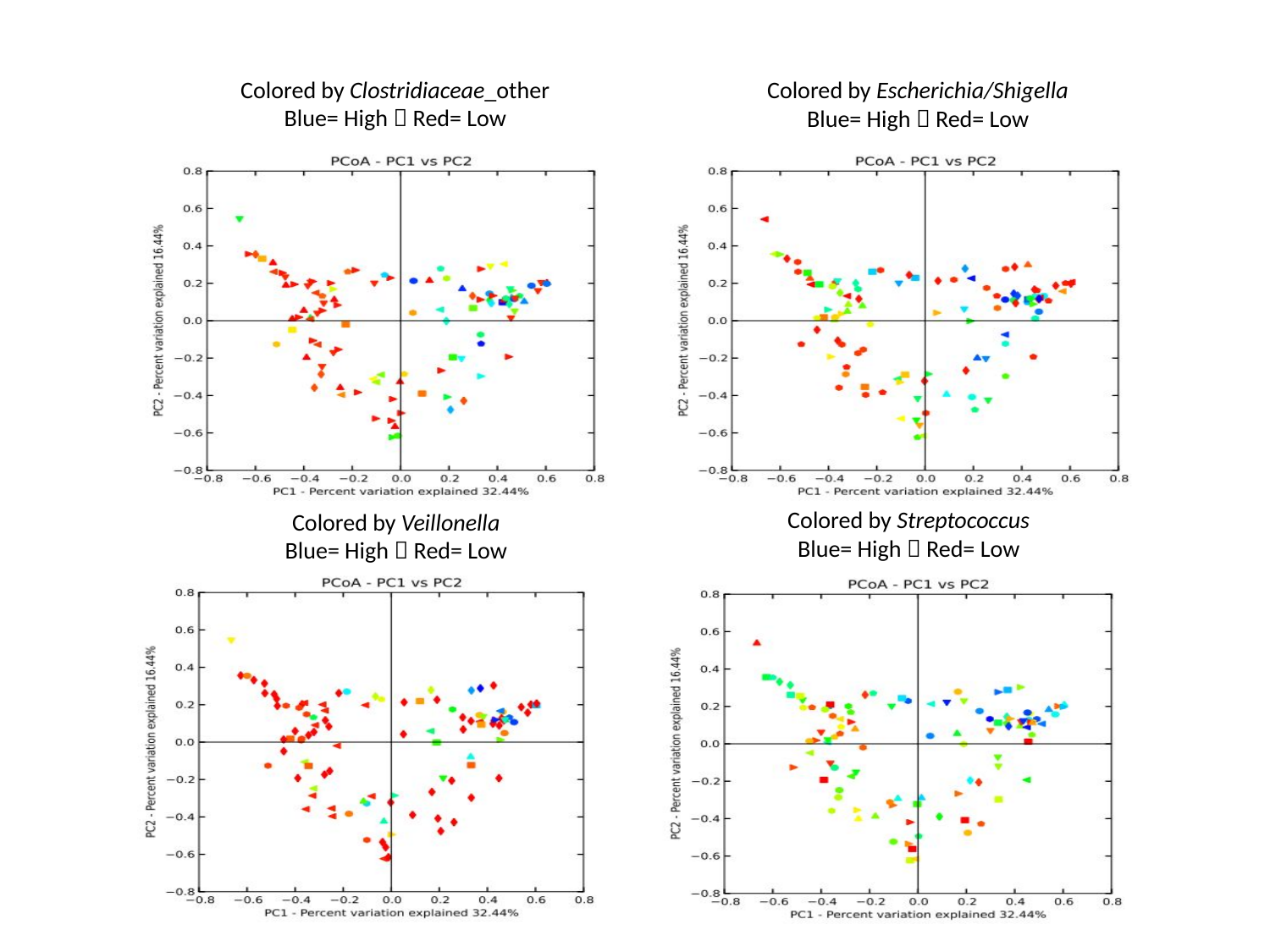

Colored by Clostridiaceae_other
Blue= High  Red= Low
Colored by Escherichia/Shigella
Blue= High  Red= Low
Colored by Streptococcus
Blue= High  Red= Low
Colored by Veillonella
Blue= High  Red= Low
