# Supplementary material for: Maternal fucosyltransferase 2 status affects the gut bifidobacterial communities of breastfed infants
Source: Microbiome. 2015 Apr 10;3:13. doi: 10.1186/s40168-015-0071-z (PMC4412032; doi:10.1186/s40168-015-0071-z)
Supplement: Additional file 9: Figure S6. — PCoA plots of the NGS data. Colored by the abundance of ‘Enterobacteriaceae_other’ (top left), Streptococcus (top right), Escherichia/Shigella (bottom left), and ‘Clostridiaceae_other’ (bottom right). Colors represent a spectrum of abundance, with blue being high and red being low. [file 40168_2015_71_MOESM9_ESM.pptx]

## Slide 1
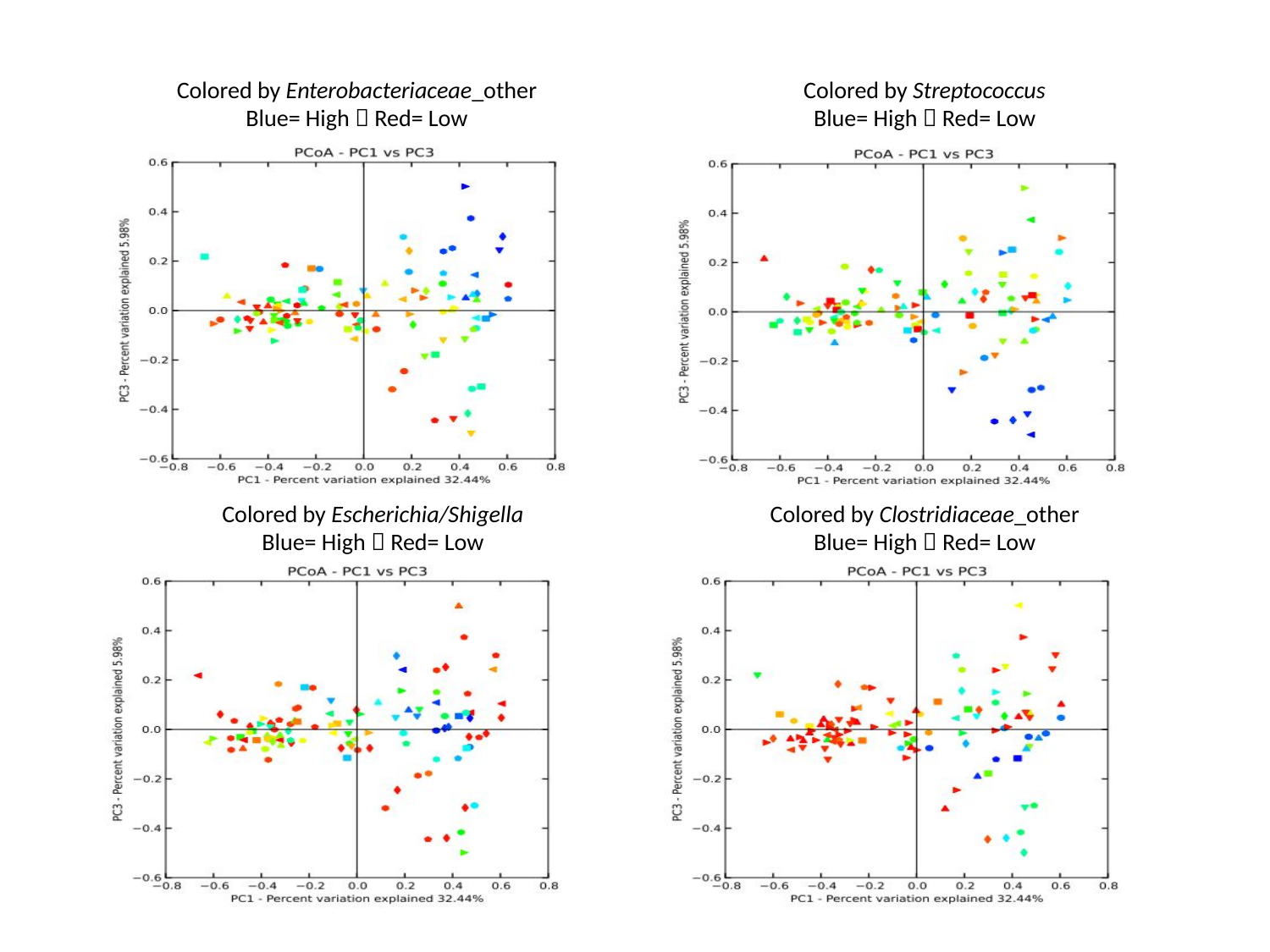

Colored by Streptococcus
Blue= High  Red= Low
Colored by Enterobacteriaceae_other
Blue= High  Red= Low
Colored by Escherichia/Shigella
Blue= High  Red= Low
Colored by Clostridiaceae_other
Blue= High  Red= Low
