# Supplementary material for: Maternal fucosyltransferase 2 status affects the gut bifidobacterial communities of breastfed infants
Source: Microbiome. 2015 Apr 10;3:13. doi: 10.1186/s40168-015-0071-z (PMC4412032; doi:10.1186/s40168-015-0071-z)
Supplement: Additional file 10: Table S4. — ANOSIM analysis. ANOSIM R statistics and p values for various ways of grouping samples. [file 40168_2015_71_MOESM10_ESM.pptx]

## Slide 1
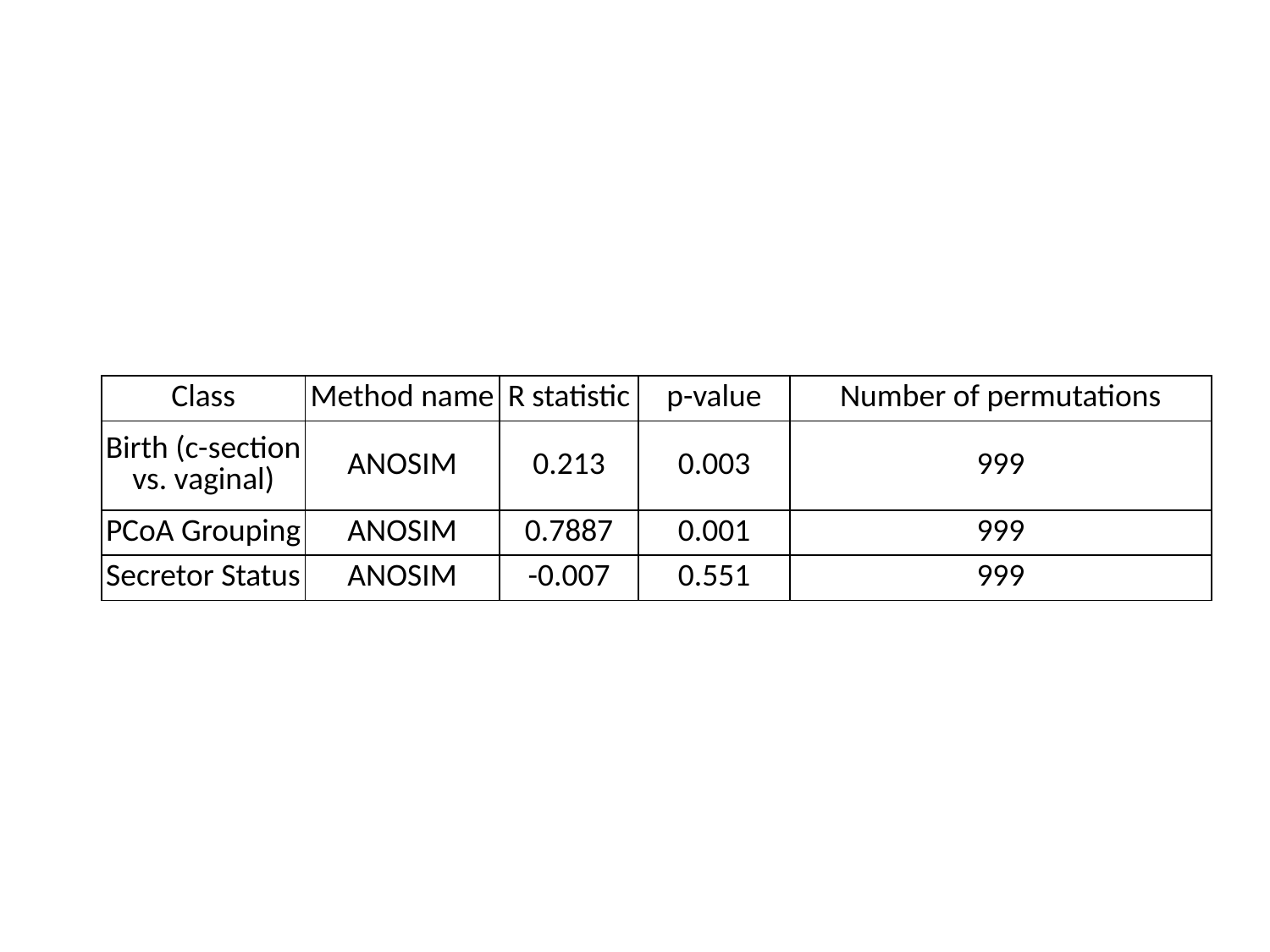

| Class | Method name | R statistic | p-value | Number of permutations |
| --- | --- | --- | --- | --- |
| Birth (c-section vs. vaginal) | ANOSIM | 0.213 | 0.003 | 999 |
| PCoA Grouping | ANOSIM | 0.7887 | 0.001 | 999 |
| Secretor Status | ANOSIM | -0.007 | 0.551 | 999 |
